# Hepatocellular Carcinoma with Gastrointestinal Involvement: A Systematic Review

**DOI:** 10.3390/diagnostics12051270

**Published:** 2022-05-19

**Authors:** Cristiana Marinela Urhut, Larisa Daniela Sandulescu, Liliana Streba, Vlad Florin Iovanescu, Sarmis Marian Sandulescu, Suzana Danoiu

**Affiliations:** 1 Department of Gastroenterology, Emergency County Hospital of Craiova, Doctoral School, University of Medicine and Pharmacy of Craiova, 200349 Craiova, Romania; cristiana.urhut@yahoo.com; 2 Department of Gastroenterology, Research Center of Gastroenterology and Hepatology, University of Medicine and Pharmacy of Craiova, 200349 Craiova, Romania; iovanescu_vlad@yahoo.com; 3 Department of Medical Oncology, Emergency County Hospital of Craiova, University of Medicine and Pharmacy of Craiova, 200349 Craiova, Romania; lilianastreba@yahoo.com; 4 Department of Surgery, Emergency County Hospital of Craiova, University of Medicine and Pharmacy of Craiova, 200349 Craiova, Romania; ssarmis@yahoo.com; 5 Department of Pathophysiology, Faculty of Medicine, University of Medicine and Pharmacy of Craiova, 200349 Craiova, Romania; suzanadanoiu@yahoo.com

**Keywords:** hepatocellular carcinoma, gastrointestinal involvement, algorithm, esophagogastroduodenoscopy, gastrointestinal bleeding

## Abstract

In this paper, we aimed to evaluate clinical and imagistic features, and also to provide a diagnostic algorithm for patients presenting with gastrointestinal involvement from hepatocellular carcinoma (HCC). We conducted a systematic search on the PubMed, Scopus and Web of Science databases to identify and collect papers oncases of HCC with gastrointestinal involvement. This search was last updated on 29 April 2022. One hundred and twenty-three articles were included, corresponding to 197 patients. The majority of the patients were male (87.30%), with a mean age of 61.21 years old. The analysis showed large HCCs located mainly in the right hepatic lobe, and highly elevated alfa-fetoprotein (mean = 15,366.18 ng/mL). The most frequent etiological factor was hepatitis B virus (38.57%). Portal vein thrombosis was present in 27.91% of cases. HCC was previously treated in most cases by transarterial chemoembolization (32.99%) and surgical resection (28.93%). Gastrointestinal lesions, developed mainly through direct invasion and hematogenous routes, were predominantly detected in the stomach and duodenum in equal measure—27.91%. Gastrointestinal bleeding was the most common presentation (49.74%). The main diagnostic tools were esophagogastroduodenoscopy (EGD) and computed tomography. The mean survival time was 7.30 months. Gastrointestinal involvement in HCC should be included in the differential diagnosis of patients with underlying HCC and gastrointestinal manifestations or pathological findings in EGD.

## 1. Introduction

Hepatocellular carcinoma (HCC) is the most common primary malignant tumor of the liver and the sixth most common cancer, according to GLOBOCAN 2020 data. Although both incidence and mortality rates declined in many high-risk areas, many patients have already reached an advanced stage at diagnosis, resulting in 830,000 deaths worldwide [1].

Hepatocellular carcinoma usually disseminates to the liver [2]. Although less common, in 30–50% of the cases, HCC can have extrahepatic spread. The most frequent areas are the lungs, followed by lymph nodes and bones [3,4,5]. Involvement of the gastrointestinal (GI) tract is a rare event, with a reported incidence of 0.5–2% of all HCCs. Higher rates of 4–12% have been recorded in autopsy cases [6,7].

Due to non-specific symptomatology or endoscopic features, GI involvement by HCC is underdiagnosed premortem [8]. The available data about this condition are provided mainly by case reports and only a few literature reviews. In 2004, Fujii, K. et al. reviewed the characteristics of 29 HCC patients with GI tract invasion [9]. Later on, in 2011, Lin, T.L. et al. assessed the course of disease and survival in 44 patients reported in the English literature with direct invasion of the GI tract by HCC [10]. In the same year, Kato, Y. et al. (2011) investigated the role of surgical treatment based on 18 cases from the literature (English literature and Japanese literature with English abstract), including his reported case [11]. In 2018, Harada, J. et al. also reviewed the clinical characteristics of esophageal metastases from HCC [12]. Recently, Yu, Y.M. et al. (2020) listed and analyzed 15 patients with metastases from HCC in the small bowel and large intestine, followed in 2021 by Mu, M. et al., who provided a literature summary of 21 HCC colonic metastases [13,14]. However, these studies included a limited number of cases and were focused on a specific route of metastasis, or the involvement of a particular segment of the GI tract. Additional cases have been reported since these previous publications.

The HCC survival rate has increased over the last three decades and we expect it to further increase as a result of improvements in therapy and early diagnosis [15]. For that reason, we also expect to see a more significant number of patients with atypical complications in our clinical practice [16]. An early diagnosis of GI involvement from HCC is a challenge for clinicians, and raising awareness of this issue is a crucial step toward it. Our systematic review complements previous studies and gives a bigger picture of the main clinical and imagistic characteristics of GI involvement from HCC. We also propose an algorithm diagnosis that would serve clinicians in making a rapid diagnosis.

## 2. Materials and Methods

A systematic review of case studies of gastrointestinal involvement from hepatocellular carcinoma was conducted according to the Preferred Reporting Items for Systematic Reviews and Meta-Analyses (PRISMA) statement (Table S1) [17].

### 2.1. Data Sources

The systematic search was performed in the PubMed, Scopus and Web of Science databases up to November 2021, with the last search update on 29 April. A search in the Scopus and Web of Science database was also performed to avoid missing relevant articles up to 29 April. Randomly discovered searches, mainly from the manual search of references, were also included.

### 2.2. Inclusion and Exclusion Criteria

Our search strategy was developed on PICO (Patient/Population, Intervention, Comparison, Outcome) elements as follows: population: patients with an imagistic or histopathological diagnosis of hepatocellular carcinoma involving the gastrointestinal tract; intervention: none; comparison: none; outcomes: case studies that reported clinical presentation, diagnosis or survival of HCC with gastrointestinal involvement. All published case reports, case series or retrospective observational studies on the PubMed database concerning GI involvement from hepatocellular carcinoma were considered eligible for inclusion. We extended our search to editorial material that provided enough data for retrieval and analysis. No restriction for the time of publication was fixed. Only articles that addressed the specific clinical topic were selected for the examination. We excluded articles written in languages other than English, those not focused on the main issue, and papers not available as full text or papers with insufficient data to support the analysis.

### 2.3. Search Strategy

The search strategy included a combination of the following terms connected with the Boolean operators “AND” and “OR”: “hepatocellular carcinoma”, “esophagus”, “stomach”, “gastric”, “duodenum”, “jejunum”, “ileum”, “intestine”, “colon”, “rectum”, “invasion”, “metastasis”. No filters were added. The search strategy is reported as Supplementary Materials (Table S2). In the first stage, duplicate references were removed from our results using EndNote 20 (Clarivate Analytics, Chandler, AZ, USA). Further, tiles/± abstracts of the records found in the first stage were screened by two reviewers, and reports that were not on the main subject were excluded. Cohen’s Kappa coefficient was calculated, obtaining a satisfactory value of 0.94. The next step was to eliminate non-English articles. The full text of the articles was retrieved when available and further analyzed. Articles containing insufficient data and those not related to the main topic were excluded.

Further, we composed a data-extracting sheet in Microsoft Excel (Microsoft Office Professional Plus 2019), Microsoft^®^ Excel^®^ 2019 MSO (Version 2111 Build 16.0.14701.20204) 32-bit. One member of our team performed the initial data extraction and it was checked for reliability by a second member.

The following data were extracted: additional bibliographic information, including name of the first author, year of publication and type of study; number of patients reported in the paper; patient’s demographics, such as gender and age; etiology; location and size of HCC; previous treatment for HCC; AFP value (ng/mL); presence of portal vein thrombosis; the involved segment of the gastrointestinal tract; clinical presentation; involvement route; imagistic methods used for diagnosis of GI lesions; endoscopic features; methods used to obtain histological specimens and whether or not immunohistochemistry was used, as well as the survival time. The extracted data were further processed. Descriptive statistics (mean, standard deviation, percentages, minimum, maximum) were used to summarize the analyzed data.

Any discrepancy between reviewers was discussed, and a final consensus was reached.

In order to assess the risk of bias in our systematic review of case reports and case series, we used the Joanna Briggs Critical Appraisal Checklist for case reports and for case series (Tables S3 and S4) [18].

## 3. Results

### 3.1. Publication Characteristics

**Figure 1:**
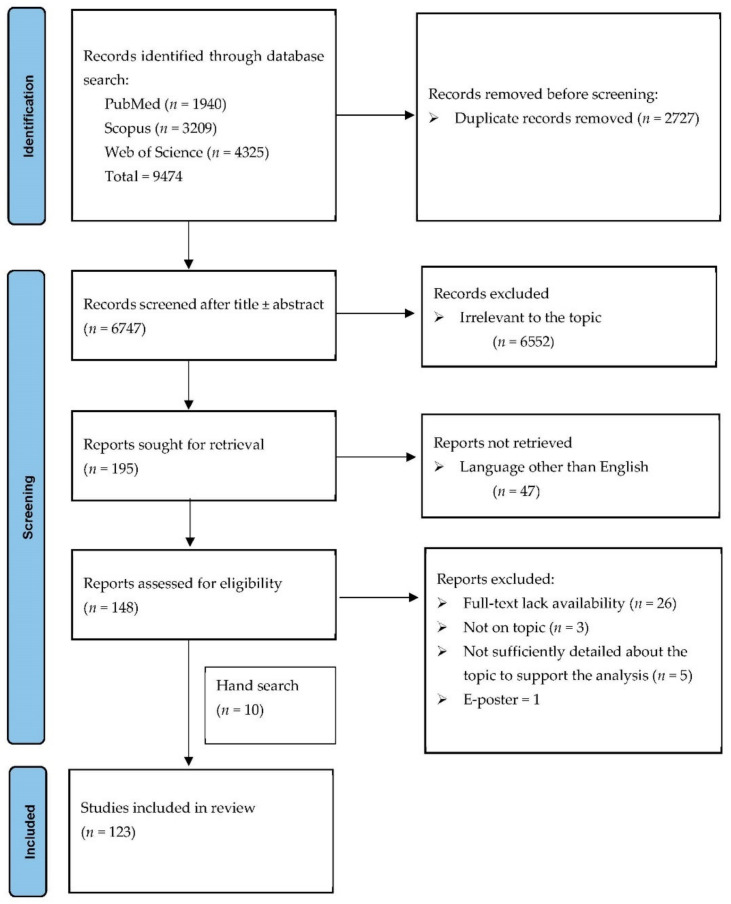
PRISMA flow diagram for the selection process of the cases.

Figure 1 illustrates the PRISMA flow diagram of our search strategy. We initially identified 9474 record titles, from which we excluded 2727 duplicates. Further, from the remaining 6747 records, only 195 were sought for retrieval. Forty-seven articles were non-English publications. In the last stage, from the 148 reports considered eligible, one was an E-poster, 26 did not have full-text availability, three were not on discussed topic and five did not have sufficient detailed data to support the analysis (Table S5). We identified ten additional publications on manual search. One hundred twenty-three articles concerning 197 patients were included in the final analysis (Table 1 and Table 2).

### 3.2. Patient Characteristics

We included 197 cases, with a mean age of 61.21 (standard deviation = 11.66), ranging from 22–86. The majority of patients were male (*n* = 172; 87.30%), with a ratio of man: woman of 6.88.

### 3.3. Etiology

Data concerning the etiology of liver disease in patients with HCC were described in 158 cases (Table 3). Hepatitis B virus (HBV) was incriminated in most patients (38.57%), followed by hepatitis C virus (HCV) (17.76%) and alcohol (7.61%). Coinfection with HBV and HCV was reported in 2.03% of cases, and only 0.5% of patients were identified with HBV, HCV, and hepatitis D virus (HVD) coinfection. Autoimmune etiology and non-alcoholic fatty liver disease (NAFLD) were suggested in one patient. In 19 cases, the underlying cause was unknown (9.64%).

### 3.4. Clinical Findings in the Study Population

A summary of the clinical characteristics of the study population is described in Table 4. Most HCCs were bulky masses, with an average tumor size of 8.66 cm (*n* = 92 hepatic nodules). Liver tumor localization was described in 158 patients. Four patients did not have any tumor recurrence at the moment of diagnosis. Hepatocellular carcinoma was located as follows: right hepatic lobe (31.47%), left hepatic lobe (21.82%), both hepatic lobes (19.28%), caudate lobe (2.03%), hepatic hilum (1.01%), peritoneum (1.01%), left hepatic lobe and caudate lobe (0.50%), right hepatic lobe and caudate lobe (0.50%) and lymph nodes (0.50%). Portal vein thrombosis was found in 27.91% (55/197) of the evaluated cases. Regarding the tumoral markers, the mean value of serum AFP was 15,366.18 ng/mL (*n* = 112 available data, 14 patients were reported as having a normal value).

### 3.5. Previous Treatment for Hepatocellular Carcinoma

Information on prior therapy for HCC is supplied in detail below in Table 5. The percentage of patients who did not receive any specific therapy was 26.90% (53/197). A relatively high number of patients were treated with TACE (32.99%, 65/197) and surgical resection (28.93%; 57/197). Among locoregional therapies, TACE was followed by: transarterial embolization (TAE) (23/197; 11.67%), radiofrequency ablation (RFA) (10. 15%, 20/197), percutaneous ethanol injection (PEI) (14/197, 7.10%) and intra-arterial chemotherapy (4/197, 2.39%). Liver transplant was performed in 5.58% of included patients. Molecular targeted therapies and systemic chemotherapy were administered to 4.56% and 5.58% of the patients, respectively. Less commonly used treatment methods were yttrium-90 radioembolization, hepatic arterial ligation, cryoablation, immunotherapy, radiotherapy and ultrasound-guided percutaneous microwave ablation.

### 3.6. Involved GI Site and Presumed Mode of Involvement

The most commonly involved sites in the gastrointestinal tract were the stomach (55/197; 27.91%) and duodenum (55/197; 27.91%,), followed by colon (32/197, 16.24%), esophagus (18/197; 8.92%), jejunum and ileum (14/197; 9.13%,) and rectum (3/197; 1.52%). Synchronous localization occurred in 9.64% of patients. The sites with concomitant involvement were: stomach and esophagus, stomach and duodenum, stomach and colon, stomach and small bowel, duodenum and colon and rectum and colon. Our study suggested that in most cases, GI involvement occurred through direct invasion (87/197; 44.16%). HCC metastasized through the hematogeneous route in 31.97% of situations (63/197). Translymphatic dissemination was reported for 3.04% (6/197); meanwhile, peritoneal spreading was found in 3.55% of patients (7/197). We also detected three cases of iatrogenically induced metastases. Moreover, both direct invasion and hematogenous spread were considered in two patients with concomitant duodenal and colon involvement and esophagus and stomach involvement, respectively. Details of the segments of the GI tract involved and the routes of involvement are listed in Table 6.

### 3.7. Clinical Presentation

GI bleeding was the most common clinical presentation (49.74%), followed by abdominal pain (26.90%), nausea and vomiting (14.72%), fecal occult blood+ (4.06%), palpable abdominal mass (3.55%) and anemia (5.07%). Among the included patients, only 2.03% of them were asymptomatic. Other less frequent clinical characteristics are summarized in Table 7.

### 3.8. Diagnosis of GI Lesions

The most frequently used diagnostic tools were upper GI endoscopy (112/197; 56.86%) and CT (112/197; 56.86%), followed by colonoscopy (24/197; 12.18%), upper GI series (18/197; 9.13%) and endoscopic ultrasound (EUS) (9/197; 4.56%). Other less frequent diagnostic methods were: magnetic resonance imaging (MRI) (7/197; 3.55%),positron emission tomography-computed tomography (PET-CT) (6/197; 3.04%), double/single-balloon enteroscopy (5/197; 2.53%), superior mesenteric angiography (4/197; 2.03), lower GI series (3/197; 1.52%), angio-CT (3/197; 1.52%), capsule endoscopy (2/197; 1.01%), scintigraphy TC-99 pyridoxyl-5-methyltryptophan (PMT) (1/197; 0.50) and celiac angiography (1/97; 0.50%). In 15 patients, the diagnosis was made either intraoperatively (9/168, 4.56%) or at autopsy (6/197; 3.04%). The full palette of the combination of diagnostic tools used is summarized in Table 8.

Endoscopic features of GI lesions are listed in Table 2. GI endoscopic procedures were used for diagnosis in 86.78% of cases (169/197). Detailed descriptions of the endoscopic aspect of the GI lesions were related in 84.61% (143/169). In two cases there was no endoscopic evidence of GI lesions. The most common endoscopic findings were: exophytic mass (15.73%), polypoid lesions (14.72%), ulcerative lesions (14.21%), submucosal tumor (8.62%), ulcer (5.58%), fistula (2.53%) and extrinsic compression (1.52%). Non-specific aspects (ulcerations, erosions etc.) were seen in 9.64% of cases.

Specimens for pathological diagnosis (*n* = 159) were obtained through endoscopic biopsies (76/197; 38.57%), surgical intervention (61/197; 30.96%), endoscopic biopsies and resected specimens (10/197, 5.07%), endoscopic ultrasound-fine needle aspiration (1/197; 0.50%) and autopsy (11/197; 5.58%). The diagnosis was not confirmed through histopathology in 15.22% of cases (30/197). Immunohistochemistry techniques were used in 26.39% of patients (52/197).

### 3.9. Prognosis of Gastrointestinal Involvement in Patients with HCC

Prognosis of patients with hepatocellular carcinoma and GI tract involvement (*n* = 158 available data; from which 12 were lost to follow-up) was dismal, with an average survival of 7.30 months. In 3.04% of cases (6/197), the diagnosis was made post-mortem at autopsy, and 1.01% (2/197) survived for less than 24 h. In the present study, only 14.72% (29/197) were still alive at the moment of the last follow-up.

## 4. Discussion

Hepatocellular carcinoma yields high recurrence rates, even after radical resection. Liver transplantation is the best treatment method because it also cures the underlying liver disease, but it is not broadly applicable [132]. However, other available therapeutic approaches, such as locoregional therapies, have been developed with the purpose of increasing the survival rate in patients with unresectable HCC [133]. Improvements in the survival period are associated with a higher risk of developing extrahepatic metastases. Among them, gastrointestinal involvement from hepatocellular carcinoma is rare [118].

In our study, both stomach and duodenum were the areas of the GI tract most frequently affected. In reviews reported in literature, the stomach was most commonly involved, followed by duodenum [10]. On the other side, esophagus metastases were very uncommon in our analysis, accounting for less than 9% of cases. Sites of the lower part of the gastrointestinal tract, such as the colon, jejunum, ileum and rectum were also less affected. In exceptional cases, a liver tumor can simultaneously involve more than one segment of the GI tract [7,51,58,59,65,66,80].

As in most reports, direct invasion was the predominant spread pattern. Factors favoring GI involvement were growth mode, size and localization of hepatic tumors [14]. Due to the the anatomical relationship between the liver and GI tract segments, HCCs localized in the right hepatic lobe tend to invade the duodenum, and those located in the left lobe usually involve the stomach [84]. The role of TACE is controversial. TACE can induce tumor adherence to the liver capsule and GI tract through necrosis and inflammation. On the other hand, HCC was diagnosed concomitant with the GI invasion in many cases, and these patients had not received any previous treatment [14]. In our analysis, TACE was previously performed in 32.99% of cases, and 26.90% did not receive any treatment.

The hematogenous route was the second most frequent path. Although it can be detected in liver cirrhosis, reverse portal flow is more frequently observed in primary hepatocellular carcinoma due to arteriovenous communications and arterial neovascularization [134]. Tumor emboli can be disseminated from the liver to the gastrointestinal tract by the hepatofugal portal flow [131]. Portal vein thrombosis is also a significant contributing element that can exacerbate the reversal of the flow [119]. This aspect is supported by the fact that 27.91% of the assessed cases in our study presented portal vein thrombosis. It is hypothesized that endoscopic therapy of esophageal varices, in particular esophageal band ligation, can promote the development of esophageal metastasis by trapping the tumoral thrombi [31]. Hiraoka, T. et al. (1986) presented two cases of hepatocellular carcinoma with invasion of the portal vein branches, in which microscopic tumoral thrombi were found in sclerosed esophageal varices [20]. Kume, K. et al. also presented a case of HCC metastases developed at the place of variceal band ligation [21].

In our review of the literature, GI tract involvement in HCC was also reported to develop after liver transplant [25,28,29,32,41,55,121]. The risk of developing uncommon metastases of HCC, including gastrointestinal metastases, can increase after a liver transplant due to a delicate physiological state or to the administration of immunosuppressive agents [32].

In exceptional cases, dissemination of tumor cells through needle track following endoscopic ultrasound-fine needle aspiration (EUS-FNA) performed for the confirmation of HCC has also been described [61]. Moreover, periampullary metastasis from HCC has been reported after biliary interventions in a patient with HCC invaded in the biliary tract [84].

Several authors also reported GI tract involvement from peritoneal spreading or lymph node metastases [8,43,55,77,80,93,101,117].

Gastrointestinal bleeding, either frank or occult, was the most common presenting feature among the studied cases. Similarly to our results, in an analysis of 30 cases with direct GI involvement from HCC, reported in the English literature, Korkolis, D.P. et al. (2009) also concluded that gastrointestinal bleeding was the main clinical presentation [46]. Besides GI bleeding, the spectrum of clinical manifestations was vast in our study results. It included abdominal pain, palpable mass, chronic anemia, dysphagia, fatigue, weight loss, nausea, vomiting, diarrhea and gastric outlet obstruction.

### Algorithm of Diagnosis

**Figure 2:**
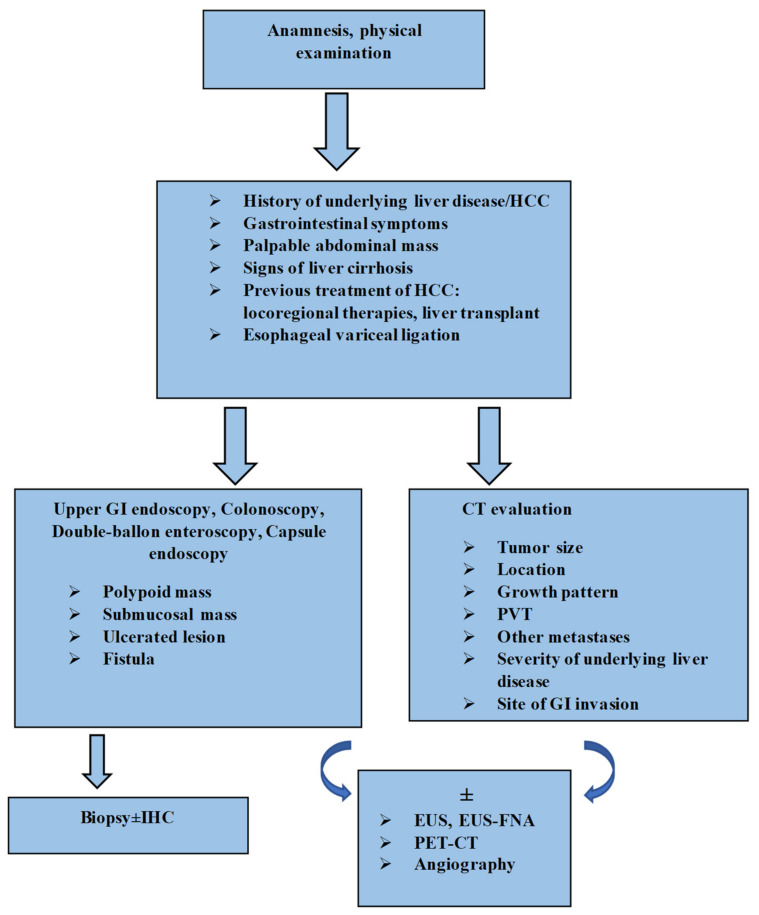
Algorithm for diagnosis of GI tract involvement from HCC. HCC: hepatocellular carcinoma; GI: gastrointestinal; CT: computed tomography; PVT: portal vein thrombosis; IHC: immunohistochemistry; EUS: endoscopic ultrasound; EUS-FNA: endoscopic ultrasound-fine needle aspiration; PET-CT: positron emission tomography-computed tomography.

On the basis of data found in the literature and the results of our research, we propose a diagnostic algorithm (Figure 2).

The diagnosis can be extremely challenging and clinicians must be aware of this condition, as early detection and prompt treatment are crucial for a better prognosis. In patients with a known clinical context of cirrhosis and hepatocellular carcinoma, with new-onset gastrointestinal symptoms, especially GI bleeding, gastrointestinal involvement from HCC should be considered as a possible etiology if other common causes are excluded [47,71]. Clinical examination plays a key role in identifying an abdominal mass [100]. In the next stage, risk-factor assessment should be conducted: tumor size, location, growth pattern, presence of portal vein thrombosis and previous locoregional therapies for the primary liver tumor, or even liver transplant and endoscopic therapy for esophageal varices [14,31,32,119]. Endoscopic examination is the standard gold method for identifying GI lesions [7]. As evidenced by the various aspects described, endoscopic features are not specific and can pose differential diagnosis problems. However, the following aspects should draw suspicion in a patient with HCC: polypoid mass, a submucosal tumor/extrinsic compression, ulcerative lesions or even the presence of a fistula [8,48,50,63,80]. The histopathologic examination is mandatory for a certain diagnosis. In some situations where there is uncertainty, investigations should include immunohistochemical tests [7,114,126]. Hepatocyte paraffin-1 (Hep par-1), glypican-3 (GPC-3), arginase-1 and polyclonal carcinoembryonicantigen (pCEA) effectively differentiate GI metastases of HCC from other types of tumors [126]. EUS and EUS-FNA are the alternative diagnostic methods for GI submucosal lesions or for when endoscopy fails to identify the tumor [33]. Radiological investigations are an excellent guidance modality. CT can describe the localization, size and extension of primary liver tumor, the status of the portal vein and lymph nodes, the site of invasion, the contiguity of HCC with the GI tract lesions and the severity of underlying liver cirrhosis, and can also exclude other metastases [8,118]. GI metastases of HCC usually display hyperenhancement in the arterial phase on CT scans, similarly to liver tumors [8]. FDG-PET/CT and angiography can complete the diagnostic workup [6,7,29,32,33,71].

Based on our reviewed articles, the mean survival was 7.30 months. Fujii, K. et al. (2004) evaluated median survival in 29 patients with HCC invading the GI tract. The estimated median survival time was 1.2 months for patients who received supportive treatment, and three months for nonsurgical treatment; meanwhile, patients treated with curative surgery showed an average survival of 9.7 months [9].

Although it may improve the clinical approach of cases with HCC and GI involvement, our study has a number of limitations. Subjectivity in data interpretation, lack of a large control group and the fact that only English-language articles were included are just some of these limitations, and may have influenced the final results.

## 5. Conclusions

In conclusion, to our knowledge, we are reporting the most extensive systematic review of case reports to date on the involvement of the gastrointestinal tract in HCC. Gastrointestinal involvement in HCC could be included in the differential diagnosis of patients with underlying HCC and gastrointestinal manifestations or pathological findings in EGD.

## Figures and Tables

**Table 1:** Literature review of cases with gastrointestinal involvement from hepatocellular carcinoma—characteristics of HCC tumors.

Author	Year	Type of Paper	Segment of GI Tract Involved	No. of Patients Reported	Age	Gender	Etiology	Localization of HCC	Dimension of HCC (cm)	Previous Treatment for HCC	PVT	AFP (ng/mL)
Sohn, D. et al. [19]	1965	Case report	esophagus	1	74	M	N/A	LHL	6	none	yes	N/A
Hiraoka, T. et al. [20]	1986	Case report	esophageal varices	2	55	M	N/A	RHL, LHL	1	none	yes	N/A
			esophageal varices		62	M	N/A	RHL	N/A	none	yes	N/A
Kume, K. et al. [21]	2000	Case report	esophagus	1	56	M	HBV	RHL, LHL	N/A	TACE	yes	12.200
Sohara, N. et al. [22]	2000	Case report	esophagus	2	54	M	HCV	RHL	N/A	TAI, PEI	yes	7820
			esophagus		56	M	UK	RHL	N/A	TAI, external beam radiotherapy	yes	990
Tsubouchi, E. et al. [23]	2005	Case report	esophagus + stomach	1	63	M	HCV	RHL, LHL	3; 2	PEI, IHAC	no	596.09
Yan, S.L. et al. [24]	2007	Editorial material	esophagus	1	53	M	HBV	LHL	N/A	None	yes	17.036
Xie, L.Y. et al. [25]	2008	Case report	esophagus	1	50	M	HBV	RHL	N/A	LT, TACE, systemic chemotherapy	yes	N/A
Choi, C.S. et al. [26]	2008	Case report	esophagus	1	66	M	UK	RHL, LHL	N/A	TACE, external beam radiotherapy	yes	3.47
Hsu, K.F. et al. [27]	2009	Editorial material	esophagus + gastric cardia	1	54	M	HBV	N/A	N/A	LT, TACE, systemic chemotherapy	no	N/A
Kahn, J. et al. [28]	2009	Letter to editor	esophagus	1	55	M	HCV	RHL, LHL	N/A	LT, TACE	yes	1426
Boonnuch, W. et al. [29]	2011	Case report	esophagus	1	59	M	N/A	No tumor reccurence	N/A	LT	no	510
Skurla, B. et al. [30]	2010	Case report	esophagus	1	56	M	alcohol	RHL, LHL	N/A (at the moment of esophageal metastasis diagnosis)	LT	yes	NR
Fukatsu, H. et al. [31]	2012	Case report	esophagus	1	63	M	NA	RHL, LHL	N/A	TACE, RFA	yes	N/A
Chen, J.X. et al. [32]	2016	Case report	esophagus	1	44	M	alcohol	NA	N/A	LT, TACE	no	17.62
Harada, J.-i. et al. [12]	2018	Case report	esophagus	1	71	M	HBV	RHL, LHL *	N/A	surgical resection	no	1800
Kongkam, P. et al. [33]	2018	Case report	esophagus	1	59	M	N/A	NA	N/A	LT	no	258.3
Boinboire, R. et al. [34]	2021	Case report	esophagus	1	66	M	alcohol	RHL, LHL	N/A	surgical resection, RFA	no	NR
Subramanian, S.K. et al. [35]	2021	Editorial material	esophagus	1	53	M	alcohol	No tumor recurrence in the liver	5; 10	systemic chemotherapy	no	NR
Shiota, T. et al. [36]	1983	Case report	stomach	1	56	M	UK	RHL, LHL	5; 10	systemic chemotherapy	no	NR
Makino, H. et al. [37]	1986	Case report	stomach	1	69	M	UK	RHL, LHL	N/A	none	yes	1,136,000
Chen, L.T. et al. [6]	1990	Retrospective analysis study	stomach	3	48	M	HBV	LHL	17	surgical resection	UK	N/A
					86	M	UK	RHL, LHL	25	none	yes	221.920
					59	M	HBV	RHL	18	TAE, IHAC	no	51.270
De Nardi, P. et al. [38]	1992	Case report	stomach	1	60	M	UK	RHL	No tumor recurrence in the liver	surgical resection	yes	24,000
Nicoll, A.J. et al. [39]	1994	Case report	stomach	1	61	M	UK	N/A	N/A	systemic chemotherapy	no	6526
Maruyama, A. et al. [40]	1999	Case report	stomach	1	65	M	HCV	RHL, LHL	N/A	TAE, IHAC, radiotherapy	no	NR
Srivastava, D.N. et al. [41]	2000	Case series	stomach	1	58	M	HCV	LHL	N/A	N/A	N/A	N/A
Wang, M.H. et al. [42]	2000	Case report	stomach	2	57	F	HBV	LHL	N/A	surgical resection, TACE	no	elevated
					58	M	HBV	LHL	N/A	surgical resection	no	N/A
Lin, C.P. et al. [7]	2000	Retrospective analysis study	stomach	5	53	M	HCV	RHL, LHL	9	none	yes	719.110
			stomach		66	M	HBV	LHL	12	surgical resection	UK	1159
			stomach		60	M	HCV	RHL, LHL	14	none	yes	136.070
			stomach		69	M	UK	RHL, LHL	14	surgical resection, TACE	no	50
			stomach		63	M	HBV	RHL, LHL	9	none	no	2432
Fujii, K. et al. [9]	2004	Case report	stomach + jejunum	1	61	M	alcohol	LHL	10; 2	none	no	19.675
Inoue, H. et al. [43]	2006	Case report	stomach	1	71	M	HCV	LHL	5	IHAC	yes	45.630
Ong, J.C.A. et al. [44]	2007	Case report	stomach	1	67	M	HBV	LHL	10	none	No	NA
Kimura, K. et al. [45]	2008	Case report	stomach	1	54	M	HBV	LHL	7.5	TAE	no	NR
Korkolis, D.P. et al. [46]	2009	Case report	stomach	1	70	M	HBV	LHL	15	none	no	2.1
Hu, M.L. et al. [47]	2009	Retrospective analysis study	stomach	7	48	M	HBV, alcohol	LHL	12	TAE	yes	969
			stomach		54	M	HBV	LHL	6	TAE	yes	>87.500
			stomach		68	M	HBV	RHL, LHL	N/A **	none	yes	440
			stomach		62	M	HBV	RHL	7	TAE	yes	2
			stomach		50	M	HBV + HCV + HDV + alcohol	RHL, LHL	N/A **	TAE	no	218
			stomach		51	M	HBV + alcohol	LHL	14	TAE	yes	6398
			stomach		71	M	HBV + alcohol	RHL, LHL	8; 6	TAE	yes	34.706
Park, H. et al. [48]	2010	Case report	stomach	1	63	M	HBV	RHL, LHL	8; 3	TACE	no	50.202
Lin, T.L. et al. [10]	2011	Case report	stomach	1	57	M	HBV	LHL	9	TAE	no	NA
Tan, W.J. et al. [49]	2013	Case report	stomach	1	76	F	cryptogenic liver cirrhosis	NA **	NA	none	no	>60.500
Sayana, H. et al. [50]	2013	Case report	stomach	1	36	M	HBV + HCV	LHL	19	TACE, sorafenib	no	7.6
Okay, E. et al. [51]	2014	Case report	stomach + transverse colon	1	44	M	HBV	LHL	28	none	no	>350.000
Inagaki, Y. et al. [52]	2014	Case report-Image of the month	stomach	1	62	M	HCV	N/A	N/A	TACE, RFA	N/A	6404
Wu, W.D. et al. [53]	2014	Case report	stomach	1	75	M	HBV	LHL	N/A	surgical resection, TACE	no	NR
Grover, I. et al. [54]	2014	Case report	stomach	1	51	M	HBV	LHL	11.5	TACE	no	elevated
Li, L. et al. [55]	2015	Case report	stomach	1	43	M	HBV	N/A	N/A	LT	no	191
Hot, S. et al. [56]	2016	Case report	stomach	1	62	M	alcohol	hepatic hilum	13	none	no	2.82
Haruki, K. et al. [57]	2016	Case report	stomach	1	73	M	UK	LHL	17	none	no	N/A
Wu, D. et al. [58]	2016	Case report	stomach + colon	1	54	M	N/A	RHL	4	surgical resection	N/A	N/A
Abdul Hakim, M.S. et al. [59]	2017	Case report	stomach + duodenum	1	73	M	N/A	RHL	N/A	RFA	no	124.800
Peng, L. et al. [60]	2018	Case report	stomach	1	22	M	HBV	RHL, LHL	8; 1.5	surgical resection	no	>1200
Kasi, M. et al. [61]	2018	Case report	stomach	1	43	M	HBV	caudate lobe	3	LT, TACE	no	69
Sakumura, M. et al. [62]	2018	Editorial material	stomach	1	68	F	HBV	N/A	N/A	TACE	yes	N/A
Bale, A. et al. [63]	2018	Editorial material	stomach	1	69	M	NAFLD	LHL	N/A	TACE	no	N/A
Imai, M. et al. [64]	2019	Case report	stomach	1	62	M	alcohol	RHL, LHL	17; 6	TACE	yes	56.388
Marques da Costa, P. et al. [65]	2019	Editorial material	stomach + duodenum	1	81	F	HCV	RHL	N/A	none	no	N/A
Kim, R. et al. [66]	2020	Case report	stomach + ascending colon	1	75	M	alcohol	N/A	N/A	surgical resection, TACE	no	NR (2.3)
Abouzied, M.M. et al. [67]	2021	Case report	stomach	1	69	M	N/A	RHL	10	surgical resection	no	NR (3.3)
Eskarous, H. et al. [68]	2022	Case report	stomach	1	82	F	NA	RHL	N/A	surgical resection	N/A	N/A
Chen, L.-T. et al. [6]	1990	Retrospective analysis study	duodenum	4	56	M	HBV	RHL	22	none	UK	3200
					56	M	UK	RHL	6	TAE	no	>700
					54	M	HBV	LHL	8	TAE, radiotherapy	yes	10
					34	M	HBV	RHL	NA	systemic chemotherapy	UK	15,435
Arima, K. et al. [69]	1992	Case report	duodenum	1	61	M	NA	RHL	3	surgical resection, systemic chemotherapy	yes	N/A
Moriura, S. et al. [70]	1995	Case report	duodenum	1	57	M	UK	hepatic hilum	7	none	no	NA
Okusaka, T. et al. [71]	1997	Case report	duodenum	1	60	M	alcohol	N/A **	11	surgical resection, TAE, PEI	no	NA
Hung, H.C. et al. [72]	1998	Case report	duodenum + stomach	1	58	M	HBV	RHL	4	surgical resection, TAE, systemic chemotherapy	no	20,799
Farrell, R. et al. [73]	1999	Case report	duodenum	1	53	M	HCV	N/A **	8	surgical resection	no	5
Srivastava, D.N. et al. [41]	2000	Case series	duodenum	1	48	M	N/A	RHL	N/A	none	N/A	N/A
Lin, C.P. et al. [7]	2000	Retrospective analysis study	duodenum	3	64	M	HBV	RHL, LHL	10	none	yes	252
			duodenum + transverse colon		67	M	HBV	RHL, LHL	15	none	yes	12,420
			duodenum		56	M	HBV	LHL	12	none	yes	<3
Del Natale, M. et al. [74]	2001	Case report	duodenum	1	67	M	alcohol	N/A	N/A	TACE	yes	24,935
Cho, A. et al. [75]	2002	Case report	duodenum	1	50	M	HBV	RHL	22	none	no	3477
Ohnishi, S. et al. [76]	2003	Letter to the editor	duodenum	1	73	M	N/A	RHL	9	surgical resection, TAE, RFA, PEI, radiotherapy	no	N/A
Uehara, K. et al. [77]	2003	Case report	duodenum	1	62	M	HCV	RHL	1	none	N/A	2000
Chung, C. et al. [78]	2009	Case report	duodenum	1	53	F	HCV, alcohol	N/A	N/A **	none	yes	NR
Kurtz, L.E. et al. [79]	2009	Editorial material	duodenum	1	78	F	HCV	RHL	8.5	RFA, sorafenib	no	N/A
Kato, Y. et al. [11]	2011	Case report	duodenum	1	63	M	UK	RHL	25; 2	none	no	848
Lin, T.L. et al. [10]	2011	Review	duodenum	1	72	M	HBV	RHL	4.5	PEI, RAE, surgical resection	no	N/A
Liang, J.D. et al. [80]	2011	Retrospective analysis study	duodenum-19 duodenum + stomach-1 duodenum + colon-1	21	62.5	M-17; F-4	HBV-12; HCV-7; HBV + HCV-2; alcohol-2	LHL, RHL-3; RHL-7; LHL-4; peritoneum-1; lymph node -1; no recurrent liver tumor = 1; NA = 4	8.6	none-4; surgical resection-3; surgical resection + TACE-7; surgical resection + TACE + PEI-1; TACE + RFA-1; TACE-4; TACE + PEI-1	yes-3; no-18	8051.6
Kim, J.N. et al. [81]	2012	Case report	duodenum	1	57	M	UK	LHL	N/A	TACE	yes	N/A
Sauer, B.G. et al. [82]	2012	Editorial material	duodenum	1	68	M	N/A	N/A	N/A	TACE, radiotherapy, systemic chemotherapy	N/A	N/A
Arima, K. et al. [83]	2015	Case report	duodenum	1	76	F	HCV	RHL	6	surgical resection	yes	34,428
Kashani, A. et al. [84]	2015	Case report	duodenum	1	62	M	HCV	N/A **	N/A	TACE	no	N/A
Lin, I.C. et al. [85]	2017	Editorial material	duodenum	1	83	M	N/A	RHL	N/A	TACE	N/A	N/A
Ito, T. et al. [86]	2019	Case report	duodenum	1	65	M	HCV	N/A	10	TACE, sorafenib	no	13,300
Liu, Y.H. et al. [87]	2020	Case report	duodenum	1	62	M	HBV	RHL	2.4	RFA, surgical resection	no	NR
Wu, Y.H. et al. [88]	2021	Case report	duodenum	1	80	F	N/A	N/A	25	none	N/A	N/A
Bonboire, R. et al. [34]	2021	Case report	duodenum	1	67	M	alcohol	RHL	79	none	no	269
Sawada, K. et al. [89]	2021	Editorial material	duodenum	1	72	M	alcohol	caudate lobe	N/A	TACE	no	N/A
Lee, Y.J. et al. [90]	2021	Retrospective analysis study	duodenum-3 stomach-1 duodenum + stomach-3	7	59.71 ***	M-7	HBV-6 UK-1	N/A	N/A	TACE, PEIT-4 TACE, RT-2	N/A	N/A
Tsujimoto, M. et al. [91]	1984	Case report	intestinal tract	1	62	M	alcohol	RHL	14	none	N/A	N/A
Chen, L.T. et al. [6]	1990	Retrospective analysis study	jejunum	1	36	M	HBV	RHL	NA	hepatic arterial ligation	UK	309
Narita, T. et al. [92]	1993	Case report	small bowel-mostly ileum + stomach	1	73	F	HBV	RHL	6	TAE	no	16,000
Tanaka, A. et al. [93]	2000	Case report	ileum	1	52	M	HBV	Peritoneum+ small intestine ****	N/A	TACE, surgical resection, systemic chemotherapy, hyperthermia	no	1160
Byun, J.R. et al. [94]	2005	Case report	ileum	1	27	M	none	RHL, caudate lobe	2.4; 3.4; 4.5	TACE	yes	6050
Kim, H.S. et al. [95]	2006	Case report	jejunum	1	65	M	HBV	N/A	N/A	none	N/A	629
Iwaki, K. et al. [96]	2008	Case report	jejunum	1	60	M	HCV	N/A	N/A	surgical resection, TACE, RFA	no	N/A *****
Choi, J.H. et al. [97]	2012	Case report	jejunum	1	54	M	HBV	N/A	N/A	sorafenib, surgical resection	yes	N/A
Kunizaki, M. et al. [98]	2012	Case report	small bowel	1	60	M	HBV	N/A	N/A	TACE, RFA	no	1345
Igawa, A. et al. [99]	2013	Case report	ileum	1	60	M	HBV	N/A	N/A	sorafenib	yes	86.5
Kanazawa, M. et al. [100]	2018	Case report	jejunum	1	76	M	alcohol	N/A	N/A	surgery, TACE, sorafenib	N/A	N/A
Shelat, V.G. et al. [101]	2018	Case report	jejunum	1	75	M	HBV	N/A	N/A	surgical resection	no	N/A
Sun, W.C. et al. [102]	2018	Editorial material	ileum	1	72	M	N/A	N/A	N/A	TACE, RFA	N/A	N/A
Mashiko, T. et al. [103]	2020	Case report	ileum	1	71	M	HBV	RHL	N/A	surgical resection, sorafenib	no	N/A
Suzuki, N. et al. [104]	2020	Case report	small bowel	1	75	M	alcohol	LHL, caudate lobe ******	2	Lenvatinib, RFA, surgical resection	no	2.2
Fukui, H. et al. [105]	1993	Case report	ascending colon	1	57	M	HCV	RHL	N/A	surgical resection, TAE	no	7
Hashimoto, M. et al. [16]	1996	Case report	transverse colon	1	72	F	HCV	RHL	4.5	TAE	no	33
Cosenza, C.A. et al. [106]	1989	Case report	duodenum (inflammatory adhesions) + ascending colon	1	82	F	HCV	RHL	NA	surgical resection, systemic chemotherapy, cryoablation	no	19
Srivastava, D.N. et al. [41]	2000	Case series	transverse colon	1	32	M	HBV	LHL	N/A	no	no	N/A
Lin, C.P. et al. [7]	2000	Retrospective analysis study	colon	3	59	M	HCV	RHL	8	TAE	yes	3319
					69	M	HBV	RHL, LHL	20	none	no	698.346
					63	M	UK	RHL, LHL	20	none	yes	46
Kurachi, K. et al. [107]	2002	Case report	colon	1	43	M	UK	LHL	12	PEIT, surgical resection	no	3
Zech, C.J. et al. [108]	2006	Case report	ascending colon	1	57	M	HBV + HCV	RHL	N/A	TACE	no	N/A
Tapuria, N. et al. [109]	2006	Case report	ascending colon	1	67	M	autoimmune cirrhosis	RHL, LHL	N/A	none	yes	20.9
Kaibori, M. et al. [110]	2007	Case report	descending colon	1	61	M	HCV	RHL, LHL	2; 1.5	TAE, PEI, surgical resection	N/A	N/A
Ng, D.S.C. et al. [111]	2007	Case report	ascending and hepatic flexure of the colon	1	35	M	HBV	RHL	12	surgical resection	no	7
Hirashita, T. et al. [112]	2008	Case report	transverse colon	2	79	M	HCV	caudate lobe	7.5	TACE	no	331
			hepatic flexure of colon		69	M	HCV	RHL	5.5	TACE, RFA	no	370
Nozaki, Y. et al. [113]	2008	Letter to the editor	ascending colon	1	69	M	N/A	LHL	N/A	surgical resection	no	686
Yoo, D.J. et al. [114]	2010	Case report	sigmoid colon	1	47	M	HBV	RHL	1.7	TACE	no	NR
Huang, S.F. et al. [115]	2011	Editorial material	rectum	1	57	F	HCV	RHL	3.8; 1.5	RFA	no	800
Shih, Y.J. et al. [116]	2012	Letter to the editor	sigmoid colon	1	50	M	UK	RHL	7; 6	none	no	NR
Haga, Y. et al. [117]	2013	Case report	cecum	1	75	F	HCV	RHL	3.8; 1.5	RFA	no	800
Sun, L.H. et al. [118]	2013	Case report	ascending colon	1	72	F	NA	caudate lobe	6	none	no	N/A
Imada, S. et al. [119]	2013	Case report	appendix	1	66	M	N/A	N/A	N/A	surgical resection, TAE	no	37
Ou, T.M. et al. [120]	2014	Case report	ascending colon + rectum	1	62	M	HBV	RHL, LHL	N/A	surgical resection, RFA, PEI, stereotactic radiosurgery, TACE	N/A	N/A
Kohli, R. et al. [121]	2014	Editorial material	splenic flexure of the colon	1	50	F	cryptogenic cirrhosis	RHL	1.5	LT, Yttrium-90 radioembolization	N/A	43
Zhu, X. et al. [122]	2016	Letter to the editor	transverse colon	1	47	M	HBV	No tumor recurrence	1.8	surgical resection, TACE, TAE	no	NR
Mitsialis, V. et al. [123]	2018	Letter to the editor	sigmoid colon	1	67	F	N/A	N/A	N/A	TACE, surgical resection	yes	N/A
Repullo, D. et al. [124]	2018	Case report	hepatic flexure of the colon	2	49	M	UK	RHL	10	none	NR	NR
Tagliabue, F. et al. [125]	2019	Case report	sigmoid colon	1	70	M	HBV	RHL	N/A	TACE	no	3
Pham, B.V. et al. [126]	2019	Case report	sigmoid colon	1	60	M	HBV	RHL, LHL	4.1	TACE	no	9.48
Soni, A. et al. [127]	2019	Case report	hepatic flexure	1	65	M	HCV	RHL, LHL	12	none	N/A	633
Yu, Y.M. et al. [13]	2020	Case report	sigmoid colon	1	60	M	HBV	RHL	N/A	resection, TACE, RFA, PRFA, sorafenib, regorafenib, immunotherapy	no	21,000
Mu, M. et al. [14]	2021	Case report	hepatic flexure	1	86	M	HBV	RHL	7	TACE, ablation	no	NR
Miyauchi, T. et al. [128]	2021	Case report	colon	1	80	M	HBV	RHL	N/A	TACE, surgical resection, RFA	no	N/A
Park, M.S. et al. [8]	2002	Retrospective analysis study	duodenum-4, colon-3, stomach-10, stomach and colon-1	18	58	M-15, F-3	N/A	11-LHL, 5-RHL, 2-LHL, RHL	mean ≈ 6	2-surgical resection, 10-none, 6-NA	yes = 10; no = 8	N/A
Liu, K.W. et al. [129]	2013	Case report	rectum	1	71	M	HBV	RHL	1.5 cm No liver tumor recurrence	RFA	no	11
Nielsen, J.A. et al. [130]	2014	Case report	rectosigmoid	1	51	M	HBV	N/A	N/A	surgical resection	no	N/A
Ikeda, A. et al. [131]	2016	Case report	rectum	1	82	F	HCV	RHL, LHL	3.5; 2.5;1	RFA, TACE	N/A	3024

F: feminine; M: masculine; HCC: hepatocellular carcinoma; PVT: portal vein thrombosis; N/A: not available; UK: unknown; HBV: hepatitis B virus; HCV: hepatitis C virus; HDV: hepatitis D virus; NAFLD: non-alcoholic fatty liver disease; LHL: left hepatic lobe; RHL: right hepatic lobe; AFP: alpha-fetoprotein; TACE: transarterial chemoembolization; TAE: transarterial embolization; RFA: radiofrequency ablation; PRFA: percutaneous radiofrequency ablation; LT: liver transplant; PEI: percutaneous ethanol injection; IHAC: intrahepatic arterial infusion chemotherapy. * Localization of HCC detected two months after esophageal metastasis diagnosis. ** Multinodular pattern of HCC. *** Mean age. **** Intraabdominal disseminated HCCs. ***** Elevated. ****** Localization of HCC recurrence.

**Table 2:** Literature review of cases with gastrointestinal involvement from hepatocellular carcinoma—features of GI metastases.

Author	Clinical Presentation	Route of Involvement	Imagistic Methods Used for the Diagnosis of GI Involvement	Endoscopic Aspect	Method of Histopathological Diagnosis	IHC	Survival Period (Months)
Sohn, D. et al. [19]	anorexia, weight loss	hematogenous, trans-lymphatic	UGI	not done	NA	no	7
Hiraoka, T. et al. [20]	hematemesis	hematogenous	autopsy	not done	autopsy	no	post-mortem diagnosis
	NA	hematogenous	autopsy	not done	autopsy	no	post-mortem diagnosis
Kume, K. et al. [21]	dysphagia, tarry tools	hematogenous	EGD, CT	polypoid lesion	autopsy	no	2
Sohara, N. et al. [22]	melena	hematogenous	EGD	submucosal tumor	EGD + autopsy	no	1
	hematemesis	hematogenous	EGD	polypoid lesion	autopsy	no	6
Tsubouchi, E. et al. [23]	epigastric discomfort	hematogenous + direct invasion	EGD, EUS, CT	polypoid lesion	EGD	yes	3
Yan, S.L. et al. [24]	melena	hematogenous	EGD	polypoid lesion	EGD	yes	1
Xie, L.Y. et al. [25]	dysphagia, odynophagia	hematogenous	EGD, CT	polypoid lesion	EGD	yes	alive at eight-month follow-up
Choi, C.S. et al. [27]	hematemesis	hematogenous	EGD, EUS	submucosal mass polypoid	EGD	yes	7
Hsu, K.F. et al. [26]	hematemesis, tarry stools	hematogenous	EGD	polypoid	EGD	yes	4
Kahn, J. et al. [28]	dysphagia	undetermined	EGD	polypoid (submucosal)	EGD	yes	9
Boonnuch, W. et al. [29]	dysphagia	hematogenous	UGI series, EGD, EUS, CT, PET-CT	extrinsic compression	resected specimen	no	N/A
Skurla, B. et al. [30]	intermittent GI bleeding, anemia	hematogenous	EGD	flat and polypoid lesions	EGD	no	alive at two-month follow-up
Fukatsu, H. et al. [31]	progressive anemia	hematogenous	EGD	polypoid/submucosal mass	EGD	yes	1
Chen, J.X. et al. [32]	nausea, abdominal discomfort, dysphagia, tarry tools	undetermined-possible translymphatic	EGD, PET-CT, CT	ulcerative mass	EGD	no	1
Harada, J. et al. [12]	asymptomatic (increased AFP)	undetermined *	EGD, CT, UGI series	polypoid lesion	EGD + resected specimen	yes	2
Kongkam, P. et al. [33]	asymptomatic **	hematogenous	EUS, PET-CT	not seen	EUS-FNA	no	20
Boinboire, R. et al. [34]	dysphagia	direct invasion from right atrium mass	EGD, CT	exophytic mass	EGD	yes	alive at fifteen-month follow-up
Subramanian, S.K. et al. [35]	hematemesis, melena	N/A	EDS, EUS	nodule	resected specimen	no	alive at five-month follow-up
Shiota, T. et al. [36]	hematemesis, melena, anasarca	direct invasion	autopsy	not done	autopsy	no	post-mortem diagnosis
Makino, H. et al. [37]	epigastralgia	hematogenous	EGD	Bormann type 2 tumor	autopsy	yes	2 months + 10 days
Chen, L.T. et al. [6]	hematemesis	undetermined	EGD	ulcerated submucosal tumor	EGD	no	1
	bloody stool	hematogenous	EGD, CT, UGI series	Borman III-like- ulcer	EGD	no	1
	fecal occult blood test+	direct invasion	EGD, CT	ulcerated submucosal tumor	EGD	no	2
De Nardi, P. et al. [38]	anorexia, weakness, weight loss, melena	hematogenous	EGD	polyps	EGD + resected specimen	yes	20
Nicoll, A.J. et al. [39]	melena, hematemesis, syncope	direct invasion	EGD	protuberant gastric nodule	resected specimen	yes	alive at seven-month follow-up
Maruyama, A. et al. [40]	melena	direct invasion	EGD, CT, UGI series	ulceration	resected specimen	no	5
Srivastava, D.N. et al. [41]	hematemesis	direct invasion	EGD, Angio-CT	ulcerative lesion	none	no	death on the same day as diagnosis
Wang, M.H. et al. [42]	tarry stools	undetermined-possible direct invasion	EGD	ulcerated, submucosal tumor	EGD	yes	N/A
	bloody sputum	undetermined-possible direct invasion	EGD	ulcerated, submucosal tumor	EGD	no	1
Lin, C.P. et al. [7]	nausea, vomiting, fecal occult blood test+	direct invasion	EGD	submucosal tumor	EGD	no	0.7
	melena, abdominal fullness	undetermined	CT	not done	none	no	9.7
	RUQ + epigastric pain	hematogenous	EGD	ulcerative tumor	EGD	no	1.8
	melena	direct invasion	EGD	submucosal tumor	EGD	no	4.7
	hematemesis, melena	direct invasion	EGD, CT	penetrated ulcer	none	no	1.6
Fujii, K. et al. [9]	anemia	direct invasion	EGD	ulcerative tumor	resected specimen	no	32
Inoue, H. et al. [43]	LUQ pain, weight loss	translymphatic (direct invasion from an enlarged lymph node)	EGD, CT	protruding, necrotic tumor	EGD	yes	NA
Ong, J.C. et al. [44]	epigastric pain, dizziness, dyspnea, GI bleeding	direct invasion	EGD	ulcer (bleeding)	resected specimen	no	alive at two-year-and-nine-month follow-up
Kimura, K. et al. [45]	progressive anemia, posprandial epigastric pressure, hematemesis	direct invasion	EGD, CT	extrinsic compression	EGD	no	2
Korkolis, D.P. et al. [46]	upper abdominal pain, gastric outlet obstruction	direct invasion	EGD, CT	protrusive, infiltrating tumor	resected specimen	no	alive at sixteen-month follow-up
Hu, M.L. et al. [47]	NA	hematogenous	EGD	ulcerative mass	EGD	no	N/A
	NA	hematogenous	EGD	ulcerative mass	EGD	no	N/A
	NA	hematogenous	EGD	ulcerative mass	EGD	no	N/A
	NA	hematogenous	EGD	ulcerative mass	EGD	no	N/A
	NA	hematogenous	EGD	ulcerative mass	EGD	no	N/A
	NA	hematogenous	EGD	ulcerative mass	EGD	no	N/A
	NA	hematogenous	EGD	ulcer with irregular margin	EGD	no	N/A
Park, H. et al. [48]	dysphagia, postprandial epigastric pain, hematemesis	direct invasion	EGD, CT	fistula	EGD	no	0.5
Lin, T.L. et al. [10]	NA	direct invasion	EGD, CT	ulcerative tumor	resected specimen	no	>80
Tan, W.J. et al. [49]	melena, hematemesis, abdominal distension, nausea, epigastric pain	direct invasion	EGD, EUS, CT	ulcer	none	no	N/A
Sayana, H. et al. [50]	hematemesis, melena	direct invasion	EGD, CT	fistula	none	no	alive at six months after diagnosis
Okay, E. et al. [51]	dyspnea, abdominal distension, nausea, vomiting, abdominal pain, fever, weight loss	direct invasion	intraoperative diagnosis	not done	resected specimen	yes	12
Inagaki, Y. et al. [52]	hematemesis	hematogenous	EGD, CT	polypoid lesions	autopsy	no	1
Wu, W.D. et al. [53]	GI bleeding	direct invasion	EGD, MRI	mass mimicking gastric cancer	resected specimen	yes	alive at twelve-month follow-up
Grover, I. et al. [54]	melena, hematemesis	direct invasion	EGD, CT	fistula	no	no	N/A
Li, L. et al. [55]	melena	translymphatic	EGD, CT	(polypoid) cauliflower like- mass	EGD	yes	4
Hot, S. et al. [56]	GI bleeding	direct invasion	EGD, CT	ulcerated mass	resected specimen + EGD	yes	<1 m
Haruki, K. et al. [57]	epigastric pain	hematogenous	EGD, CT, MRI	submucosal tumor	resected specimen	no	alive at thirteen-month follow-up
Wu, D. et al. [58]	melena, anemia	undetermined	EGD, colonoscopy	N/A	resected specimen	no	50
Abdul Hakim, M.S. et al. [59]	anemia, melena	hematogenous	EGD	fungating, nodular mass	EGD	yes	1
Peng, L. et al. [60]	hepatalgia, asthenia	undetermined *	EGD, CT	protrusion like stromal tumor	resected specimen	yes	alive at six-month follow-up
Kasi, M. et al. [61]	anemia	needle track seeding (EUS)	EGD, MRI, PET-CT	polypoid, ulcerated mass	EGD + resected specimen	yes	N/A
Sakumura, M. et al. [62]	anemia, leg numbness	hematogenous	EGD	polyp	EGD	yes	N/A
Bale, A. et al. [63]	upper GI bleeding	undetermined	EGD, CT	fistula	none	no	N/A
Imai, M. et al. [64]	anemia	hematogenous	EGD	elevated lesion	EGD	yes	5
Marques da Costa, P. et al. [65]	abdominal pain, melena	direct invasion	EGD, CT	lobulated mass	EGD	yes	<1 (0.75)
Kim, R. et al. [66]	dyspnea, melena	hematogenous	EGD, CT, colonoscopy	fungating mass-stomach; ulcerofungating tumor—ascending colon	EGD + colonoscopy+ surgical resection	yes	1.5
Abouzied, M.M. et al. [67]	weakness, anemia	probably hematogenous	MRI, EGD, PET-CT	polyps	EGD	no	alive at 15-month follow-up
Eskarous, H. et al. [68]	dysphagia	N/A	EGD	polyps	EGD	yes	N/A
Chen, L.T. et al. [6]	nausea, vomiting, fecal occult blood test+	direct invason	EGD	polypoid (cauliflower tumor)	EGD	no	<1 (0.75)
	melena	direct invasion	EGD, celiac angiography, CT	ulcerated, submucosal tumor	none	no	1
	epigastric pain, fecal occult blood test+	direct invasion	EGD, UGI series,	penetrating ulcer	none	no	4
	melena	direct invasion	EGD, CT, UGI series	polypoid (cauliflower tumor)	EGD	no	2
Arima, K. et al. [69]	hematemesis, melena	hematogenous	EGD	Bormann 2 type elevation with large tumor	EGD + autopsy	no	17
Moriura, S. et al. [70]	anemia	direct invasion	EGD, UGI series	ulcer	EGD + resected specimen	no	alive at 22-month follow-up
Okusaka, T. et al. [71]	GI bleeding, abdominal pain	direct invasion	autopsy	duodenum not analysed at EGD	autopsy	no	post-mortem diagnosis
Hung, H.-C. et al. [72]	abdominal pain, tarry stools	direct invasion	EGD, CT	ulcerative mass	EGD	no	6
Farell, R. et al. [73]	GI bleeding, lethargy	direct invasion	EGD, EUS	persistent nodular ulcer	EGD-not suggestive	no	N/A
Srivastava, D.N. et al. [41]	GI bleeding	direct invasion	EGD	ulcerative mass	EGD	no	2
Lin, C.P. et al. [7]	RUQ pain, fecal occult blood test+	hematogenous	EGD	ulcerative tumor	EGD	no	2.2
	RUQ pain, fecal occult blood test+	direct invasion	CT	not done	none	no	1.5
	GI bleeding	direct invasion	EGD, CT	ulcerative tumor	EGD	no	3
Del Natale, M. et al. [74]	abdominal pain, asthenia, dyspnea, anemia	direct invasion	EGD, CT	fistula	no	no	N/A
Cho, A. et al. [75]	palpable abdominal tumor, vomiting	direct invasion	CT, EGD	submucosal tumor	resected specimen	no	N/A
Ohnishi, S. et al. [76]	hematemesis	direct invasion	CT, EGD, UGI series	obstruction by the invading tumor	autopsy	no	2
Uehara, K. et al. [77]	no symptoms described	compression of a lymph node metastasis	CT, upper roentgenography	normal duodenal mucosa	none	no	Alive—no signs of recurrence at 22-month follow-up
Chung, C. et al. [78]	melena, abdominal pain	undetermined *	EGD	ulcerative tumor + nodule resembeling liver parenchyma	EGD	yes	7
Kurtz, L.E. et al. [79]	melena, anemia	direct invasion	EGD. CT	infiltrating mass	none	no	N/A
Kato, Y. et al. [11]	painful epigastric mass	direct invasion	UGI series	not done	resected specimen	yes	8
Liang, J.D. et al. [80]	GI bleeding-17, abdominal pain-2, anemia-1	direct invasion-14; undetermined-1; metastases-6, (hematogeneous/translymphatic-5, peritoneal spreading-1)	CT-4; EGD-4; intraoperative diagnosis-1; CT + EGD-12; UGI series-2	ulceration-13; tumor mass-10; fistula-1	EGD-2; resected specimen-7; none-12	no	mean 10.5
Lin, T.L. et al. [10]	tarry stools	direct invasion	CT, EGD	ulcerative mass	resected specimen	no	>68
Kim, J.N. et al. [81]	melena, dyspnea	direct invasion	CT, EGD	protrusive mass	EGD	no	3
Sauer, B.G. et al. [82]	GI bleeding, nausea, vomiting	direct invasion	EGD, CT	large mass (liver) penetrating the pyloric channel causing gastric obstruction	EGD	yes	1
Arima, K. et al. [83]	N/A	hematogenous	CTHA, CTAP	not done	resected specimen	no	N/A
Kashani, A. et al. [84]	fatigue, GI bleeding	spread of HCC tumoral cells after biliary interventions	EGD, MRI	periampullary mass	EGD	yes	few months
Lin, I.C. et al. [85]	melena	direct invasion	EGD, CT	mass	EGD	no	N/A
Ito, T. et al. [86]	anemia	direct invasion	EGD, CT	ulcerative lesion	EGD + resected specimen	no	alive at three-year follow-up
Liu, Y.H. et al. [87]	hematemesis, tarry stools	direct invasion	EGD, CT	ulcer	resected specimen	no	alive at seven-year follow-up
Wu, Y.H. et al. [88]	tarry stools	direct invasion	EGD, EUS	ulcerative mass	EGD	yes	N/A
Bonboire, R. et al. [34]	melena	direct invasion	EGD, abdominal arteriography, CT	submucosal mass	no	no	6
Sawada, K. et al. [89]	hematemesis	direct invasion	EGD, CT	ulcer-ulcerative lesion-submucosal tumor-like ulcer	EGD	yes	7.5
Lee, Y.J. et al. [90]	nausea, vomiting, dysphagia	direct invasion-4 translymphatic-extraluminal compression due to metastatic lymph nodes/3	EGD	ulcerative mass-4 submucosal tumor-3	N/A	N/A	<2 months
Tsujimoto, M. et al. [91]	abdominal pain, vomiting, abdominal fullness	hematogenous	autopsy	not done	autopsy	yes	post-mortem diagnosis
Chen, L.T. et al. [6]	melena	hematogenous	superior mesenteric angiography	not done	laparotomy	no	0.5
Narita, T. et al. [92]	N/A	hematogenous	autopsy	not done	autopsy	no	post-mortem diagnosis
Tanaka, A. et al. [93]	increased AFP, palpable mass	peritoneal spread	intraoperative diagnosis	not done	resected specimen	no	15
Byun, J.R. et al. [94]	dysuria, fecaluria	peritoneal spread	CT, barium study	not done	resected specimen	no	N/A
Kim, H.S. et al. [95]	abdominal pain, nausea, vomiting	hematogenous	CT, US, intraoperative diagnosis	not done	resected specimen	yes	N/A
Iwaki, K. et al. [96]	asymptomatic	hematogenous	intraoperative diagnosis	not done	resected specimen	yes	alive at twenty-one months
Choi, J.H. et al. [97]	abdominal pain, abdominal distension	hematogenous	intraoperative diagnosis	not done	resected specimen	no	1
Kunizaki, M. et al. [98]	fatigue, anemia, melena	hematogenous	double-balloon enteroscopy	protruding lesion	double balloon enteroscopy + resected specimen	yes	N/A
Igawa, A. et al. [99]	melena, anemia	hematogenous	capsule endoscopy, double-balloon enteroscopy	polypoid lesion	double-balloon enteroscopy	yes	2
Kanazawa, M. et al. [100]	melena, light-headedness	undetermined	capsule endoscopy, double-balloon enteroscopy	mass lesion	double-balloon enteroscopy	yes	0.5
Shelat, V.G. et al. [101]	abdominal pain, vomiting, diarrhea	peritoneal spreading	CT	not done	resected specimen	yes	alive at eight-month follow-up
Sun, W.C. et al. [102]	melena	metastasis ***	single balloon retrograde enteroscopy	protrusive mass	single-balloon enteroscopy	no	N/A
Mashiko, T. et al. [103]	abdominal pain, vomiting	hematogenous	CT	not done	resected specimen	yes	alive at eighty-two-month follow-up
Suzuki, N. et al. [104]	abdominal pain	hematogenous	intraoperative diagnosis	not done	resected specimen	no	alive at two-month follow-up
Fukui, H. et al. [105]	asymptomatic	possible hematogenous	CT, colonoscopy, scintigraphy Tc-99 MPT	elevated lesion	colonoscopy	no	N/A
Hashimoto, M. et al. [16]	melena	direct invasion	colonoscopy, lower GI series superior mesenteric angiography	ulcerations	colonoscopy + resected specimen	no	N/A
Cosenza, C.A. et al. [106]	weakness, fatigue, rectorrhagia	duodenum-direct invasion, colon-hematogenous	colonoscopy, lower GI series	polypoid mass	colonoscopy	no	N/A
Srivastava, D.N. et al. [41]	bloody stools	direct invasion	angio-CT	not done	none	no	0.75
Lin, C.P. et al. [7]	bloody stools	direct invasion	colonoscopy, CT	polypod tumor	colonoscopy	no	1.2
Lin, C.P. et al. [7]	epigastric pain, fecal occult blood test+	direct invasion	CT, superior mesenteric angiography	not done	none	no	4.7
Lin, C.P. et al. [7]	bloody stools	direct invasion	CT, superior mesenteric angiography	not seen	none	no	4
Kurachi, K. et al. [107]	epigastric discomfort	peritoneal spread	intraoperative diagnosis	not done	resected specimen	no	alive at five-year-and-nine-month follow-up
Zech, C.J. et al. [108]	abdominal pain, fever, hemorrhagic diarrhea	direct invasion	CT, colonoscopy	inflammatory mucosal lesions	resected specimen	no	N/A
Tapuria, N. et al. [109]	rectorrhagia, anemia	hematogenous	CT, colonoscopy	obstructing tumor	colonoscopy	yes	few months
Kaibori, M. et al. [110]	melena	metastasis ***	intraoperative diagnosis	not done	resected specimen	no	5
Ng, D.S.C. et al. [111]	rectorrhagia	hematogenous	colonoscopy	fungating tumor	colonoscopy + resected specimen	no	alive at more than five years
Hirashita, T. et al. [112]	epigastric pain	direct invasion	CT	not done	resected specimen	no	6
	melena, abdominal distension	direct invasion	colonoscopy, CT	lobulated tumor	resected specimen	no	1
Nozaki, Y. et al. [113]	abdominal pain, hematochezia	metastasis ***	colonoscopy	erosive tumor lesion	colonoscopy	no	1
Yoo, D.J. et al. [114]	abdominal pain	hematogenous	colonoscopy, CT	bulging mass	resected specimen	yes	alive at four-month follow-up
Huang, S.F. et al. [115]	bloody stools	metastasis ***	colonoscopy	soft-tissue-like lesion	resected specimen	yes	N/A
Shih, Y.J. et al. [116]	abdominal pain, fever	hematogenous	CT	not done	resected specimen	no	6
Haga, Y. et al. [117]	abdominal pain, vomiting	peritoneal spreading	CT	not seen	resected specimen	no	N/A
Sun, L.H. et al. [118]	abdominal pain	hematogenous	CT	not done	resected specimen	yes	8
Imada, S. et al. [119]	asymptomatic	hematogenous	US, CT, MRI, CT, PET-CT	normal aspect	resected specimen	yes	alive at 20-month follow-up
Ou, T.M. et al. [120]	tenesmus	hematogenous	colonoscopy	polyps	resected specimen	no	1
Kohli, R. et al. [121]	hematochezia	hematogenous	colonoscopy	friable, necrotic lesion	colonoscopy	no	N/A
Zhu, X. et al. [122]	fecal occult blood test+	hematogenous	colonoscopy	mass	resected specimen	yes	12
Mitsialis, V. et al. [123]	abdominal pain, diarrhea, hematochezia	hematogenous	colonoscopy	ulceration	resected specimen	no	N/A
Repullo, D. et al. [124]	abdominal pain, fever, weight loss	direct invasion	colonoscopy, CT		resected specimen	no	N/A
Tagliabue, F. et al. [125]	GI bleeding	hematogenous	colonoscopy, CT	mass	resected specimen	yes	N/A
Pham, B.V. et al. [126]	tenesmus, abdominal pain	hematogenous	colonoscopy, CT	mass	resected specimen	yes	N/A
Soni, A. et al. [127]	rectorrhagia, anemia	direct invasion	colonoscopy, CT	ulcerated lesion	colonoscopy	no	at diagnosis
YU, Y.M. et al. [13]	hematochezia	hematogenous	colonoscopy, CT	protuberant mass	resected specimen	yes	alive at three-month follow-up
Mu, M. et al. [14]	abdominal pain, nausea, vomiting	direct invasion	MRI	not seen	resected specimen	no	10
Miyauchi, T. et al. [128]	abdominal pain, fever	hematogenous	CT	not done	resected specimen	no	30
Park, M.S. et al. [8]	GI bleeding-3; lower GI bleeding-1; epigastric discomfort (pain, nausea, vomiting)-9, palpable mass-5	direct invasion-12; hematogenous-3; undetermined-2; peritoneal spreading-1	CT-13, CT + UGI series-5, endoscopy = 13	N/A	EGD-13; resected specimen-5	no	lost to follow up- 12 patients; 2 months- 3 patients; alive- 3 patients.
Liu, K.W. et al. [129]	tenesmus	direct seeding after RFA	CT	not seen	resected specimen	yes	19
Nielsen, J.A. et al. [130]	abdominal pain, diarrhea	hematogenous	colonoscopy	mass	colonoscopy	yes	N/A
Ikeda, A. et al. [131]	bloody stools	hematogenous	colonoscopy, lower GI series, CT	protruding tumor	resected specimen, colonoscopy-not conclusive	yes	5

HCC: hepatocellular carcinoma; N/A: not available; RUQ: right upper quadrant; LUQ: left upper quadrant; GI: gastroenterology; CT: computed tomography; EGD: esophagogastroduodenoscopy; IHC: immunohistochemistry; UGI series: upper gastrointestinal series; US: ultrasound; EUS: endoscopic ultrasound; EUS-FNA: endoscopic ultrasound-fine needle aspiration; PET-CT: positron emission tomography-computed tomography; MRI: magnetic resonance imaging; CTHA: computed tomography angiography; CTAP: computed tomography arterial portography; Tc-99 m: technetium-99 m; PMT: pyridoxyl-5-methyltryptophan. * possible hematogenous. ** surveillance post-liver transplant. *** the specific route of metastasis was not clarified.

**Table 3:** Etiology of liver disease in patients with hepatocellular carcinoma.

Risk Factor	*n* (%)
HBV	76 (38.57%)
HCV	35 (17.76%)
Alcohol	15 (7.61%)
HBV + HCV	4 (2.03%)
HBV, Alcohol	3 (1.52%)
HCV, Alcohol	1 (0.50%)
HBV + HCV + HVD + Alcohol	1 (0.50%)
NAFLD	1 (0.50%)
Autoimmune	1 (0.50%)
Cryptogenic	2 (1.01%)
Unknown	19 (9.64%)
Not specified	39 (23.35%)

HBV: hepatitis B virus; HCV: hepatitis C virus; HVD: hepatitis D virus; NAFLD: non-alcoholic fatty liver disease.

**Table 4:** Summary of clinical characteristics of study patients.

Size of HCC (*n* = 92 Hepatic Nodules)	[Mean ± SD] 8.66 ± 6. 22 cm
**Localization of HCC (*n* = 158)**	
RHL	62 (31.47%)
LHL	43 (21.82%)
LHL, RHL	38 (19.28%)
Caudate lobe	4 (2.03%)
Peritoneum	2 (1.01%)
Lymph nodes	1 (0.50%)
LHL, caudate lobe	1 (0.50%)
RHL, caudate lobe	1 (0.50%)
Hepatic hilum	2 (1.01%)
No tumor recurrence	4 (2.03%)
**Portal vein thrombosis**	
Present	55 (27.91%)
Absent	109 (55.32%)
Not available	33 (16.75%)
**AFP**	Mean = 15,366.18 ng/mL

RHL: right hepatic lobe; LHL: left hepatic lobe; AFP: alfa-fetoprotein.

**Table 5:** Previous treatment of HCC.

Methods of Treatment	*n* (%)
TACE	65 (32.99%)
Surgical resection	57 (28.93%)
TAE	23 (11.67%)
Radiofrequency ablation	20 (10.15%)
Liver transplant	11 (5.58%)
Systemic chemotherapy	11 (5.58%)
Targeted molecular therapies	9 (4.56%)
Percutaneous ethanol injection	14 (7.10%)
Radiotherapy	8 (4.06%)
Intra-arterial chemotherapy	4 (2.03%)
Immunotherapy	1 (0.50%)
Yttrium-90 radioembolization	1 (0.50%)
Hepatic arterial ligation	1 (0.50%)
Ultrasound guided percutaneous microwave ablation	1 (0.50%)
Crioablation	1 (0.50%)
None	53 (26.90%)
N/A	7 (3.55%)

TACE: transarterial chemoembolization; TAE: transarterial embolization; N/A: not available.

**Table 6:** Involved GI site and involvement route.

GI Site Involved by HCC (*n* = 197)	*n* (%)
Stomach	55 (27.91%)
Duodenum	55 (27.91%)
Colon	32 (16.24%)
Esophagus	18 (8.92%)
Small bowel	14 (9.13%)
Esophaghus + Stomach	2 (1.01%)
Stomach + colon	4 (2.03%)
Stomach + duodenum	7 (3.55%)
Stomach + small bowel	2 (1.01%)
Duodenum + Colon	3 (1.52%)
Rectum	3 (1.52%)
Rectosigmoid	1 (0.50%)
Rectum and colon	1 (0.50%)
**Involvement route**	
Direct invasion	87 (44.16%)
Hematogenous route	63 (31.97%)
Translymphatic route	6 (3.04%)
Peritoneal spreading	7 (3.55%)
Iatrogenic	3 (1.52%)
Direct invasion + Hematogenous route	2 (1.19%)
Hematogenous + translymphatic route	1 (0.50%)
Metastasis (hematogenous or translymphatic)	17 (8.62%)
Undetermined	9 (4.56%)
N/A	2 (1.19%)

**Table 7:** Clinical features of included patients.

Symptom	*n* (%)
GI bleeding	98 (49.74%)
Abdominal pain	53 (26.90%)
Nausea/Vomiting	29 (14.72%)
Dysphagia	15 (7.61%)
Anemia	10 (5.07%)
Fecal occult blood+	8 (4.06%)
Gatric outlet obstruction	8 (0.50%)
Palpable abdominal mass	7 (3.55%)
Weight loss	5 (2.53%)
Dyspnea	5 (2.53%)
Abdominal distension	5 (2.53%)
Fever	5 (2.53%)
Fatigue	4 (2.03%)
Diarrhea	4 (2.03%)
Abdominal fulness	2 (1.01%)
Tenesmus	2 (1.01%)
Anorexia	1 (0.50%)
Anasarca	1 (0.50%)
Syncope	1 (0.50%)
Dizziness	1 (0.50%)
Asymptomatic	4 (2.03%)
Not available	11 (5.58%)

**Table 8:** Palette of diagnostic tools used for GI lesion.

Diagnostics Tools	*n* (%)
Upper GI endoscopy	47 (23.85%)
Upper GI endoscopy, CT	55 (27.91%)
CT	13 (6.59%)
Lower GI endoscopy, CT	10 (5.07%)
Lower GI endoscopy	8 (4.06%)
CT, upper GI series	7 (3.55%)
Upper GI endsocopy, CT, upper GI series	5 (2.53%)
Upper GI endoscopy, EUS	4 (2.03%)
Upper GI series	4 (2.03%)
Double baloon/single baloon enteroscopy	3 (1.52%)
CT, superior mesenteric angiography	2 (1.01%)
Capsule endoscopy + double ballon enteroscopy	2 (1.01%)
Upper GI endoscopy, MRI, PET-CT	2 (1.01%)
Lower GI endoscopy, lower GI series	2 (1.01%)
Upper GI endoscopy, EUS, CT	2 (1.01%)
Upper GI endoscopy, MRI	2 (1.01%)
Upper GI endoscopy, upper GI series	2 (1.01%)
Upper GI series, upper GI endoscopy, EUS, CT, PET-CT	1 (0.50%)
EUS, PET-CT	1 (0.50%)
Upper GI endoscopy, MRI, CT	1 (0.50%)
MRI	1 (0.59%)
Upper GI endoscopy, lower GI endoscopy, CT	1 (0.50%)
Upper GI endoscopy, lower GI endoscopy	1 (0.50%)
Upper GI endsoscopy, celiac angiography, CT	1 (0.50%)
Upper GI endoscopy, CTA	1 (0.50%)
CT, CTA	1 (0.50%)
CTA	1 (0.59%)
CT, lower GI endoscopy + Tc-99 m PMT Scintigraphy	1 (0.50%)
CT, lower GI endsocopy, lower GI series	1 (0.50%)
Lower GI endoscopy, lower GI series, superior mesenteric angiography	1 (0.50%)
Superior mesenteric angiography	1 (0.50%)

GI: gastroenterology; CT: computed tomography; EUS: endoscopic ultrasound; PET-CT: positron emission tomography-computed tomography; MRI-magnetic resonance imaging; CTA: computed tomography angiography; Tc-99 m: technetium-99 m; PMT: pyridoxyl-5-methyltryptophan.

## Data Availability

Data supporting the findings of this study are available within the article and its Supplementary Materials.

## Supplementary Materials

The following supporting information can be downloaded at: https://www.mdpi.com/article/10.3390/diagnostics12051270/s1. Table S1: PRISMA 2020 checklist. Table S2: Complete search strategy. Table S3: Methodological quality of included case reports using JBI Critical Appraisal Checklist for case reports. Table S4: Methodological quality of included case reports using JBI Critical Appraisal Checklist for case series. Table S5: Relevant studies excluded from the systematic review.
