# Supplementary material for: Hepatocellular Carcinoma with Gastrointestinal Involvement: A Systematic Review
Source: Diagnostics (Basel). 2022 May 19;12(5):1270. doi: 10.3390/diagnostics12051270 (PMC9140172; doi:10.3390/diagnostics12051270)
Supplement: Supplementary file 1 [file diagnostics-12-01270-s001.zip › Supplementary file 2-Complete search strategy.pdf]

Table S2. Complete search strategy

Name of databse	Search strategy	Number of records
PubMed	(("hepatocellular carcinoma") AND (esophagus* OR stomach* OR gastric* OR duodenum* OR jejunum* OR ileum* OR small bowel* OR colon* OR rectum*) AND (invasion* OR metastasis*))	1940
Scopus	TITLE-ABS-KEY ("hepatocellular carcinoma") AND TITLE-ABS-KEY (esophagus* OR stomach* OR gastric* OR duodenum* OR jejunum* OR ileum* OR "small bowel*" OR colon* OR rectum*) AND TITLE-ABS-KEY (invasion* OR metastasis*)	3209
Web of Science	ALL=("hepatocellular carcinoma*") AND ALL=(esophagus* OR stomach* OR gastric* OR duodenum* OR jejunum* OR ileum* OR small bowel* OR colon* OR rectum*) AND ALL=(invasion* OR metastasis*)	4325
