# Supplementary material for: Hepatocellular Carcinoma with Gastrointestinal Involvement: A Systematic Review
Source: Diagnostics (Basel). 2022 May 19;12(5):1270. doi: 10.3390/diagnostics12051270 (PMC9140172; doi:10.3390/diagnostics12051270)
Supplement: Supplementary file 1 [file diagnostics-12-01270-s001.zip › Supplementary file 3-4 Risk of bias assesment.pdf]

Risk of bias was assessed by the Joanna Briggs Insitute critical appraisal tools for case reports and case series.

A. JBI Critical Appraisal Checlist for case reports.

Q1. Were patient's demographic characteristics clearly described?

Q2. Was the patient's history clearly described and presented as a timeline?

Q3. Was the current clinical condition of the patient on presentation clearly described?

Q4. Were diagnostic tests or assessment methods and the results clearly described?

Q5. Was the intervention(s) or treatment procedure(s) clearly described?

Q6. Was the post-intervention clinical condition clearly described?

Q7. Were adverse events (harms) or unanticipated events identified and described?

Q8. Does the case report provide takeaway lessons?

B. JBI Critical Appraisal Checklist for case series

Q1. Were there clear criteria for inclusion in the case series?

Q 2. Was the condition measured in a standard, reliable way for all participants included in the case series?

q3. Were valid methods used for identification of the condition for all participants included in the case series?

Q 4. Did the case series have consecutive inclusion of participants?

Q 5. Did the case series have complete inclusion of participants?

Q 6. Was there clear reporting of the demographics of the participants in the study?

Q7. Was there clear reporting of clinical information of the participants?

Q 8. Were the outcomes or follow up results of cases clearly reported?

Q 9. Was there clear reporting of the presenting site(s)/clinic(s) demographic information?

Q 10. Was statistical analysis appropriate?

Note: Moola S, Munn Z, Tufanaru C, Aromataris E, Sears K, Sfetcu R, Currie M, Qureshi R, Mattis P, Lisy K, Mu P-F. Chapter 7: Systematic reviews of etiology and risk. In: Aromataris E, Munn Z (Editors). Joanna Briggs Institute Reviewer's Manual. The Joanna Briggs Institute, 2017. Available

https://jbi.global/critical-appraisal-tools

Table S3. Methodological quality of included case reports using JBI Critical Appraisal Checklist for case reports.

Author	Q1	Q2	Q3	Q4	Q5	Q6	Q7	Q8
Sohn D et al. [19]	yes	yes	yes	unclear	N/A	no	yes	yes
Hiraoka T et al [20]	yes	No	yes	yes	yes	no	yes	yes
	yes	unclear	no	yes	yes	yes	yes	yes
Kume K et al [21]	yes	yes	yes	yes	N/A	N/A	yes	yes
Sohara N et al. [22]	yes	yes	yes	yes	N/A	N/A	yes	yes
	yes	yes	yes	yes	yes	yes	yes	yes
Tsubouchi E [23]	yes	yes	yes	yes	N/A	N/A	yes	yes
Yan SL et al [24]	yes	unclear	yes	yes	yes	yes	yes	yes
Xie LY [25]	yes	yes	yes	yes	yes	yes	yes	yes
Choi CS et al [26]	yes	yes	yes	yes	unclear	no	yes	yes
Hsu KF et al. [27]	yes	yes	yes	yes	unclear	unclear	yes	yes
Kahn J et al [28]	yes	yes	yes	yes	yes	no	yes	yes
Boonnuch W et al. [29]	yes	unclear	yes	yes	yes	yes	yes	yes
Skurla B et al. [30]	yes	yes	yes	yes	yes	unclear	yes	yes
Fukatsu H [31]	yes	yes	unclear	yes	yes	unclear	yes	yes
Chen JX et al [32]	yes	yes	yes	yes	N/A	N/A	yes	yes
Harada JL et al [12]	yes	yes	yes	yes	yes	yes	yes	yes
Kongkam P et al [33]	yes	no	no	yes	no	no	yes	yes

Boinboire R et al. [34]	yes	yes	yes	yes	yes	yes	yes	yes
	yes	yes	yes	yes	yes	yes	yes	yes
Subramanian SK et al. [35]	yes	yes	unclear	yes	yes	no	yes	yes
Shiota T et al [36]	yes	yes	yes	yes	N/A	N/A	yes	yes
Makino H et al. [37]	yes	no	yes	yes	yes	unclear	yes	yes
De Nardi P et al . [38]	yes	yes	yes	yes	yes	yes	yes	yes
Nicoll AJ et al [39]	yes	yes	yes	yes	yes	yes	yes	yes
Maruyama A et al. [40]	yes	yes	yes	yes	yes	yes	yes	yes
Srivastava DN et al [41]	yes	no	yes	yes	yes	yes	yes	yes
Srivastava DN et al [41]	yes	no	no	yes	yes	yes	yes	yes
Srivastava DN et al [341]	yes	unclear	unclear	yes	yes	yes	yes	yes
Wang MH et al. [37]	yes	yes	yes	yes	yes	no	yes	yes
Wang MH et al. [42]	yes	yes	yes	yes	N/A	N/A	yes	yes
Fujii K et al. [9]	yes	yes	unclear	yes	yes	yes	yes	yes
Inoue H et al. [43]	yes	yes	yes	yes	yes	yes	yes	yes
Ong JC et al. [44]	yes	yes	yes	yes	yes	yes	yes	yes
Kimura K et al [45]	yes	yes	yes	yes	yes	yes	yes	yes
Korkolis DP et al. [46]	yes	yes	yes	yes	yes	yes	yes	yes
Park H et al. [48]	yes	yes	yes	yes	yes	yes	yes	yes
Tan WJ et al. [49]	yes	yes	yes	yes	yes	yes	yes	yes
Sayana H et al [50]	yes	yes	yes	yes	yes	yes	yes	yes

Okay E et al. [51]	yes	unclear	unclear	yes	yes	yes	yes	yes
Inagaki Y et al. [52]	yes	yes	unclear	yes	yes	unclear	yes	yes
Wu WD et al. [53]	yes	yes	yes	yes	yes	no	yes	yes
Grover I et al. [54]	yes	yes	yes	yes	yes	no	yes	yes
Li L et al. [55]	yes	yes	yes	yes	yes	unclear	yes	yes
Hot S et al. [56]	yes	unclear	yes	yes	yes	yes	yes	yes
Haruki K et al. [57]	yes	no	unclear	yes	yes	yes	yes	yes
Wu D et al. [58]	yes	unclear	yes	unclear	yes	no	yes	yes
Abdul Hakim MS et al. [59]	yes	yes	yes	yes	yes	yes	yes	yes
Peng L et al. [60]	yes	yes	yes	yes	yes	yes	yes	yes
Kasi M et al. [61]	yes	yes	unclear	yes	yes	yes	yes	yes
Sakumura M et al. [62]	yes	yes	unclear	yes	unclear	no	yes	yes
Bale A et al. [63]	yes	no	unclear	yes	N/A	N/A	yes	yes
Imai M et al. [64]	yes	unclear	unclear	yes	yes	yes	yes	yes
Marques da Costa P et al. [65]	yes	unclear	yes	yes	yes	unclear	yes	yes
Kim R et al. [66]	yes	yes	yes	yes	yes	yes	yes	yes
Abouzied MM et al. [67]	yes	yes	yes	yes	yes	unclear	yes	yes
Eskarous H et al. [68]	yes	yes	unclear	yes	no	no	yes	yes
Arima K et al. [69]	yes	yes	yes	yes	yes	unclear	yes	yes
Moriura S et al. [70]	yes	yes	unclear	yes	yes	yes	yes	yes
Okusaka T et al. [71]	yes	unclear	yes	yes	yes	yes	yes	yes

Hung HC et al. [72]	yes	yes	yes	yes	yes	yes	yes	yes
Farell R et al. [73]	yes	yes	yes	yes	yes	yes	yes	yes
Del Natale M et al. [74]	yes	yes	yes	yes	no	N/A	yes	yes
Cho A et al. [75]	yes	unclear	yes	yes	yes	yes	yes	yes
Ohnishi S et al. [76]	yes	yes	yes	yes	yes	yes	yes	yes
Uehara K et al. [77]	yes	unclear	yes	yes	yes	yes	yes	yes
Chung C et al. [78]	yes	yes	yes	yes	yes	no	yes	yes
Kurtz LE et al. [79]	yes	yes	unclear	yes	yes	no	yes	yes
Lin TL et al. [10]	yes	yes	unclear	yes	yes	yes	yes	yes
	yes	yes	unclear	yes	yes	yes	yes	yes
Kato Y et al [11]	yes	unclear	unclear	yes	yes	yes	yes	no
Kim JN et al. [81]	yes	yes	yes	yes	yes	yes	yes	yes
Sauer BG ey al. [82]	yes	yes	unclear	yes	yes	unclear	yes	yes
Arima K et al [83]	yes	unclear	no	yes	yes	no	yes	yes
Kashani A et al. [84]	yes	yes	unclear	yes	yes	unclear	yes	yes
Lin IC [85]	yes	yes	unclear	yes	no	no	yes	yes
Ito T et al. [86]	yes	yes	yes	yes	yes	yes	yes	yes
Liu YH et al. [87]	yes	yes	unclear	yes	yes	yes	yes	yes
Wu Yh ET alL. [88]	yes	unclear	yes	yes	unclear	unclear	yes	yes
Sawada et al. [89]	yes	unclear	unclear	yes	yes	unclear	yes	yes
Tsuijomoto M et al. [91]	yes	yes	yes	yes	no	no	yes	yes
Narita T et al. [92]	yes	yes	no	yes	unclear	no	yes	yes

Tanaka A et al. [93]	yes	yes	yes	yes	yes	yes	yes	yes
Byun JR et al. [94]	yes	yes	yes	yes	yes	yes	no	yes
Kim HS et al. [95]	yes	yes	yes	yes	yes	no	yes	yes
Iwaki K et al. [96]	yes	yes	yes	yes	yes	yes	yes	yes
Choi JH et al. [97]	yes	yes	yes	yes	yes	yes	yes	yes
Kunizaki M et al. [98]	yes	yes	unclear	yes	yes	no	yes	yes
Igawa A et al. [99]	yes	unclear	yes	yes	yes	yes	yes	yes
Kanazawa M et al. [100]	yes	unclear	yes	yes	no	no	yes	yes
Shelat VG et al. [101]	yes	yes	yes	yes	yes	yes	yes	yes
Sun WC et al. [102]	yes	unclear	unclear	yes	no	unclear	yes	yes
Mashiko T e al. [103]	yes	yes	yes	yes	yes	yes	yes	yes
Suzuki N et al. [104]	yes	yes	yes	yes	yes	yes	yes	yes
Fukui H et al. [105]	yes	yes	yes	yes	no	no	yes	yes
Hashimoto M et al. [16]	yes	yes	yes	yes	yes	unclear	yes	yes
Cosenza CA et al. [106]	yes	yes	yes	yes	yes	yes	yes	yes
Kurachi K et al. [107]	yes	yes	yes	yes	yes	yes	yes	yes
Zech CJ et al. [108]	yes	yes	yes	yes	yes	no	yes	yes
Tapuria N et al. [109]	yes	yes	yes	yes	yes	no	yes	yes
Kaibori M et al. [110]	yes	yes	unclear	yes	yes	no	yes	yes
Ng DSC et al .[111]	yes	yes	yes	yes	yes	yes	yes	yes

Hirashita T et al. [112]	yes	yes	unclear	yes	yes	yes	yes	yes
	yes	yes	unclear	yes	yes	yes	yes	yes
Nozaki Y et al. [113]	yes	yes	unclear	yes	no	no	yes	yes
Yoo DJ et al. [114]	yes	yes	unclear	yes	yes	no	yes	yes
Huang SF et al. [115]	yes	yes	no	yes	yes	no	yes	yes
Shih YJ et al. [116]	yes	no	yes	yes	yes	no	yes	yes
Haga Y et al. [117]	yes	yes	yes	yes	yes	yes	yes	yes
Sun LH et al. [118]	yes	yes	yes	yes	yes	yes	yes	yes
Imada S et al. [119]	yes	yes	yes	yes	yes	yes	yes	yes
Ou TM et al. [120]	yes	yes	unclear	yes	yes	unclear	yes	yes
Kohli R et al. [121]	yes	yes	yes	yes	yes	unclear	yes	yes
Zhu X et al. [122]	yes	yes	yes	yes	yes	yes	yes	yes
Mitsialis V et al. [123]	yes	yes	yes	yes	yes	no	yes	yes
Repullo D et al. [124]	yes	yes	yes	yes	yes	yes	yes	yes
Tagliabue F et al. [125]	yes	unclear	yes	yes	yes	yes	yes	yes
Pham BV et al. [126]	yes	yes	yes	yes	yes	no	yes	yes
Soni A et al. [127]	yes	yes	yes	yes	no	no	yes	yes
YU YM et al. [13]	yes	yes	yes	yes	yes	yes	yes	yes
Mu M et al. [14]	yes	yes	yes	yes	yes	yes	yes	yes
Miyauchi et al .[128]	yes	yes	yes	yes	yes	yes	yes	yes

Liu KW et al. [129]	yes	yes	yes	yes	yes	yes	yes	yes
Nielsen JA et al. [130]	yes	unclear	yes	yes	yes	unclear	yes	yes
Ikeda A [131]	yes	yes	no	yes	yes	yes	yes	yes

*NA-not applicable

Table S4. Methodological quality of included case series using JBI Critical Appraisal Checklist for case series.

Author	Q1	Q2	Q3	Q4	Q5	Q6	Q7	Q8	Q9	Q10
Chen LT et al. [6]	yes	yes	yes	yes	yes	yes	yes	yes	yes	yes
Lin CP et al. [7]	yes	yes	yes	unclear	unclear	yes	yes	yes	yes	yes
Park MS et al. [8]	yes	yes	yes	yes	no	yes	yes	unclear	yes	yes
HU ML et al. [47]	yes	yes	yes	unclear	yes	yes	yes	no	yes	yes
Liang JD et al. [80]	yes	yes	yes	yes	yes	yes	yes	yes	yes	yes
Liu YJ et al. [90]	yes	yes	yes	yes	yes	yes	no	yes	yes	yes

*N/A-not applicable
