# Supplementary material for: Hepatocellular Carcinoma with Gastrointestinal Involvement: A Systematic Review
Source: Diagnostics (Basel). 2022 May 19;12(5):1270. doi: 10.3390/diagnostics12051270 (PMC9140172; doi:10.3390/diagnostics12051270)
Supplement: Supplementary file 1 [file diagnostics-12-01270-s001.zip › Supplementary file 5-List of relevant studies excluded from the review.pdf]

Table S5. Relevant studies excluded from the systematic review

No	Reference	Reason for exclusion
1	Lynch P, L, Jordan PH, Graham DY. Hepatocellular carcinoma metastatic to the stomach presenting as bleeding multiple craterogenic ulcers. Am J Gastroenterol. 1989 Jun;84(6):653-	Full-text not available
2	Oka Y, Okazaki N, Hirota T et al. Metastatic Hepatocellular Carcinoma Mimicking an Early Gastric Cancer: A Case Report, *Japanese Journal of Clinical Oncology*, Volume 9, Issue 1, June 1979, Pages 189–197.	Full-text not available
3	Ryu JK, Lee SB, Kim KH, Yoh KT. Surgical treatment in a patient with multiple implanted intraperitoneal metastases after resection of ruptured large hepatocellular carcinoma. Hepatogastroenterology. 2004 Jan-Feb;51(55):239-42	Full-text not available
4	Cho A, Ryu M, Yoshinaga Y, Ishikawa Y, Miyazawa Y, Okazumi S, Ochiai T. Hepatocellular carcinoma with unusual metastasis to the esophagus. Hepatogastroenterology. 2003 Jul-Aug;50(52):1143-5.	Full-text not available
5	Sano T, Izuishi K, Takebayashi R, Akamoto S, Kakinoki K, Okano K, Masaki T, Suzuki Y. Surgical approach for extrahepatic metastasis of HCC in the abdominal cavity. Hepatogastroenterology. 2011 Nov-Dec;58(112):2067-70.	Full-text not available
6	Tsai C.-C., Shiue J.-W., Lai K.-H., Wen S.-C., Liu Y.-F., Lee T.-M. (1997) Hepatocellular carcinoma invading the stomach presented as gastrointestinal bleeding: Report of a case. Gastroenterological Journal of Taiwan, 14 (1), pp. 43 - 49	Full-text not available
7	Yang, H., Yeh, T. , Jan, Y., Jeng, L. Chen, M. (2001). Hepatocellular carcinoma presenting with gastrointestinal bleeding. Formosan Journal of Surgery, 34(4), 198-202	Full-text not available
8	Yoshikawa, I., Murata, I., Tabaru, A., Otsuki, M. (1994). Metastatic hepatocellular carcinoma of the stomach presenting as a bleeding polypoid lesion. Digestive Endoscopy, 6(3), 248-252.	Full-text not available
9	Yoshida H, Onda M, Tajiri T, et al. Successful surgical treatment of peritoneal dissemination of hepatocellular carcinoma. Hepato-gastroenterology. 2002 Nov-Dec;49(48):1663-1665.	Full-text not available
10	Monica Lobo, MD, Zhenggang Xiong, Victoria Collins, A Rare Hepatocellular Carcinoma Metastasis to the Esophagus: A Case Report and Literature Review, *American Journal of Clinical Pathology*, Volume 150, Issue suppl_1, October 2018, Page S13,	Full-text not available

11	Perini MV, Herman P, Pessoa R, Saad WA, D'Albuquerque LA. Unusual cause of gastrointestinal bleeding in a cirrhotic patient: hepatocellular carcinoma with gastric invasion. Hepatobiliary Pancreat Dis Int. 2009 Aug;8(4):431-3.	Full-text not available
12	Green PH, Fevre DI, Barratt PJ. Metastatic hepatoma in the stomach masquerading as a leiomyoma. Aust N Z J Med. 1976 Aug;6(4):341-4. doi: 10.1111/imj.1976.6.4.341.	Full-text not available
13	Perini MV, Herman P, Pessoa R, Saad WA, D'Albuquerque LA. Unusual cause of gastrointestinal bleeding in a cirrhotic patient: hepatocellular carcinoma with gastric invasion. Hepatobiliary Pancreat Dis Int. 2009 Aug;8(4):431-3.	Full-text not available
14	Reyes del Pozo E. "Retrograde" metastasis of primary carcinoma of the liver to the stomach. J Am Med Womens Assoc. 1969 May;24(5):423..	Full-text not available
15	Perrakis, A., Croner, R., Weidinger, T., Zopf, S., Küfner, M., Hohenberger, W., & Müller, V. (2012). Surgical treatment of advanced hepatocellular carcinoma with biliary and portal tumor thrombosis and invasion of the colon. Surgical Chronicles, 17(1), 45-48.	Full-text not available
16	Terada T, Maruo H. Unusual extrahepatic metastatic sites from hepatocellular carcinoma. Int J Clin Exp Pathol. 2013 Apr 15;6(5):816-20.	Not sufficiently detailed to support the analysis
17	Yamada K, Tohyama H, Shizawa Y, Kohno M, Fukunishi Y, Tomoe M. Direct duodenal invasion of hepatocellular carcinoma. Intestinal hemorrhage treated by transcatheter arterial embolization. Clin Imaging. 1998 May-Jun;22(3):196-9. doi: 10.1016/s0899-7071(97)00125-3.	Full-text not available
18	Hatano E, Ikai I, Shimizu M, Maetani Y, Konda Y, Chiba T, Terajima H, Yamamoto N, Yamamoto Y, Shimahara Y, Yamaoka Y. Resection for hepatocellular carcinoma with duodenal invasion: report of a case. Hepatogastroenterology. 2003 Jul-Aug;50(52):1034-6.	Full-text not available
19	Hung GU, Yeh YH, Chen YL, Lee JK. Duodenal metastasis from hepatocellular carcinoma demonstrated on FDG PET/CT imaging. Clin Nucl Med. 2008 Dec;33(12):859-60. doi: 10.1097/RLU.0b013e31818c8cfe.	Full-text not available
20	Rana SS, Behera A, Kalra N, Bhasin DK. Hepatocellular carcinoma infiltrating the duodenum presenting as hematemesis. Indian J Gastroenterol. 2008 Nov-Dec;27(6):253.	Full-text not available
21	Kanematsu M, Hoshi H, Goto H, Kajiura Y. Duodenal bulb involvement from metastasized porta-hepatis lymph node of hepatocellular carcinoma. AJR Am J Roentgenol. 1997 Jan;168(1):280-1. doi: 10.2214/ajr.168.1.8976965.	Full-text not available
22	Yang PM, Sheu JC, Yang TH, Chen DS, Yu JY, Lee CS, Hsu HC, Sung JL. Metastasis of hepatocellular carcinoma to the proximal jejunum manifested by occult gastrointestinal bleeding. Am J Gastroenterol. 1987 Feb;82(2):165-7.	Full-text not available

23	Yeh CN, Chen HM, Chen MF, Chao TC. Peritoneal implanted hepatocellular carcinoma with rupture after TACE presented as acute appendicitis. Hepatogastroenterology. 2002 Jul-Aug;49(46):938-40.	Full-text not available
24	Chou HS, Lee KF, Yeh CN, Chen MF, Jeng LB. Long-term survival following resection of peritoneal implantation from hepatocellular carcinoma: a case report. Hepatogastroenterology. 2005 Jul-Aug;52(64):1221-3.	Full-text not available
25	Yoo SW, Kim DY, Lee C, Min JJ, Kwon SY. Small Bowel Metastasis From Hepatocellular Carcinoma Detected by 18F-FDG PET/CT But Not by 11C-Acetate PET/CT. Clin Nucl Med. 2017 Dec;42(12):966-967. doi:10.1097/RLU.0000000000001882.	Full-text not available
26	Chen CY, Lu CL, Pan CC, Chiang JH, Chang FY, Lee SD. Lower gastrointestinal bleeding from a hepatocellular carcinoma invading the colon. J Clin Gastroenterol. 1997 Jul;25(1):373-5. doi: 10.1097/00004836-199707000-00019.	Full-text not available
27	Hu J, Hu C. Thoracic vertebral metastasis from progressive hepatocellular carcinoma following liver transplantation combined with resection of mesenteric and colonic metastases: A case report. Medicine (Baltimore). 2020 Oct 30;99(44):e22937. doi: 10.1097/MD.0000000000022937.	Not sufficiently detailed to support the analysis
28	Uenishi T, Kubo S, Hirohashi K, Hamba H, Tanaka H, Shuto T, Yamamoto T, Kinoshita H. Successful surgical control for hepatocellular carcinoma disseminated to the peritoneum: a case report. Hepatogastroenterology. 2002 Mar-Apr;49(44):532-4.	Full-text not available
29	Lee, S. K. and M. J. Song (2017). "Drop metastasis from hepatocellular carcinoma after percutaneous radiofrequency ablation therapy." Korean Journal of Internal Medicine 32(5): 943-944.	
30	Yamamoto H, Watanabe K, Nagata M, Yano Y, Akai T, Honda I, Watanabe S, Soda H, Matsuzaki O. Transformation of fibrolamellar carcinoma to common hepatocellular carcinoma in the recurrent lesions of the rectum and the residual liver: a case report. Jpn J Clin Oncol. 1999 Sep;29(9):445-7. doi: 10.1093/jjco/29.9.445. Erratum in: Jpn J Clin Oncol 1999 Nov;29(11):593.	Fibrolamellar carcinoma-not on topic
31	Shigemori M, Kondo M, Azechi H, Inoue F, Tamura J, Kobayashi H, Saiga T. A case of ectopic hepatocellular carcinoma in the jejunum. J Gastroenterol. 2006 Sep;41(9):913-8. doi: 10.1007/s00535-006-1872-4.	Ectopic HCC in the jejunum<br>No full text available<br>Not on topic
32	Arakawa M, Kage M, Matsumoto S, Akagi Y, Noda T, Fukuda K, Nakashima T, Okuda K. Frequency and significance of tumor thrombi in esophageal varices in hepatocellular carcinoma associated with cirrhosis. Hepatology. 1986 May-Jun;6(3):419-22. doi: 10.1002/hep.1840060316. PMID: 3011630.	Not sufficiently detailed to support the analysis
33	Fang, J. Z., et al. (2021). "Surgical Treatment of Postoperative Abdominal Metastases of Hepatocellular Carcinoma: 10-Year Experience in a Single Center." Cancer Manag Res 13: 8673-8683.	Not sufficiently detailed to support the analysis

34	Ofosu, A., et al. (2019). "Metastatic Hepatocellular Carcinoma into the Transverse Colon." Journal of Gastrointestinal Cancer 50(3): 644-646.	Not sufficiently detailed to support the analysis
35	Barrett, L., et al. (2020). "Hepatocellular Carcinoma With Metastasis to the Gastrojejunal Anastomosis of a Gastric Bypass Site." American Journal of Gastroenterology 115: S1415-S1416. Doar abstract	E-poster
